# Multidomain DNA–Protein Mining Reveals Polymorphic Variations in RhlB Enhancing Monorhamnolipid Biosynthesis

**DOI:** 10.1021/acssynbio.5c00828

**Published:** 2026-04-17

**Authors:** Pavlos Trus, Chien-Yi Chang

**Affiliations:** † School of Dental Sciences, Faculty of Medical Sciences, 5994 Newcastle University, Newcastle Upon Tyne NE2 4BW, UK; ‡ Biosciences Institute, Faculty of Medical Sciences, Newcastle University, Newcastle Upon Tyne NE2 4HH, UK

**Keywords:** rhamnolipid, biosurfactant, pseudomonas, DNA−protein mining

## Abstract

Rhamnolipids are glycolipid biosurfactants produced by *Pseudomonas aeruginosa*, valued for their surface activity, biodegradability, and potential as green alternatives to synthetic surfactants. The first rhamnosylation step, catalyzed by RhlB, is central to the catalytic efficiency of monorhamnolipid formation and subsequent dirhamnolipid biosynthesis. To explore natural diversity and enhance RhlB activity, we established a multilayer DNA–protein mining pipeline to identify polymorphisms influencing catalytic activity. The framework integrated analyses of nucleotide transitions/transversions, mutation patterns, binding-site configurations, stability hotspots, and active-site plasticity. Phylogenetic analysis revealed two hypermutator phylogroups, highlighting evolutionary divergence within RhlB. Three *rhlB* variants (*rhlB1*, *rhlB2*, *rhlB3*) were experimentally validated in a modular *rhlAB* genetic construct for monorhamnolipid biosynthesis in *Escherichia coli*. Using a defined synthetic medium and high-throughput screening platform, monorhamnolipid production was quantified as 20.1 μg mL^–1^ for the reference *rhlAB* construct, 22.18 μg mL^–1^ for *rhlAB*1, 3.2 μg mL^–1^ for the dysfunctional *rhlAB*2, and 55.51 μg mL^–1^ for *rhlAB*3, representing a 2.76-fold increase over the reference. Functional analysis suggested that mutation H263R in RhlB2 disrupted catalytic activity, while reversion (R263H) restored activity to 24.63 μg mL^–1^. Enhanced catalysis in RhlB3 was attributed to loop mutations (I234V and P238R) that improved substrate binding affinity and domain alignment. This study demonstrates that integrating computational mining with experimental validation can reveal naturally optimized enzyme variants, providing a scalable framework for accelerating enzyme engineering and advancing sustainable biosurfactant production.

## Graphical abstract

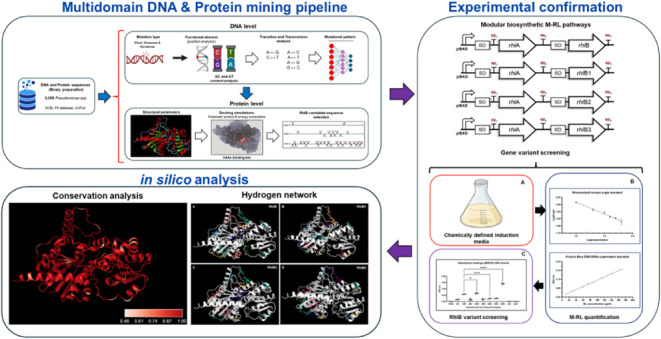

## Introduction

One of the major challenges in the field of enzyme engineering is understanding how polymorphic variations impact an enzyme’s structure and catalytic activity.^1^ Directed evolution experiments have demonstrated that only a small fraction (around 5%) of mutations improve functionality,^2–4^ while most (95%) are either detrimental or neutral. Importantly, beneficial mutations can occur throughout the entire protein sequence and not just within the active site.^5^ Identifying these key regions for improvement remains an arduous undertaking, as functionally important changes vary between enzymes. Beneficial mutations have been found in active sites, binding pockets, and the protein surface.^2,6,7^ Naturally occurring enzymes with enhanced function often exhibit greater genetic variability at both the nucleotide and protein levels.^8^

Conformational dynamics are also recognized as crucial for enzymatic catalysis.^9–11^ These dynamics involve three phases: a transition state preceding the reaction, an excited state facilitating the reaction, and a relaxation phase returning to the stable conformation. Mutations creating new residues within substrate/product binding sites can enhance conformational dynamics by stabilizing transition and excited states, increasing catalytic activity and product release.^11,12^ Unlike biochemical reactions that transpire on the picosecond-scale, conformational changes are cumulative events occurring over nanoseconds to microseconds. They exhibit non-Markovian behavior with previous interactions affecting folding and unfolding kinetics.^13^ These movements arise from fluctuations in backbone or side-chain torsion angles.^14^ Mutations within active-site loops can anchor proteins into an open and noncatalytic conformation.^15,16^ Studies have shown that spatially adjacent mutations can enhance structural stability by altering the hydrophobic core network, while surface mutations can influence reaction rates through free energy changes within the catalytic site.^17^ Conversely, reduced plasticity of active-site loops or the formation of new amino acid interactions can decrease substrate binding efficiency.^18,19^

Amino acid side chains modulate protein dynamics through alterations in flexibility, hydrogen/hydrophobic networks and can impact binding affinity when located within active sites.^14^ Interestingly, beneficial mutations failed to improve function when tested individually; however, deleterious mutations when combined with distal (>20 Å) mutations demonstrated a negation in effect.^20,21^ Mutations evolve independently but can also influence subsequent integration.^20^ Synergistic mutations have been shown to enhance catalytic activity and stabilize new interactions.^22^ Cannon and Reynolds demonstrated this synergistic effect by optimizing subtilisin enzymes through site-directed mutagenesis, achieving a remarkable 830-fold and 184-fold increase in reaction rate for *Bacillus subtilis* and *B. pumilus*, respectively.^23^ Despite these advances, identifying functionally important segments remains a key challenge in enzyme engineering.^24^

Microbial enzyme diversity within a single genus or even among closely related strains reflects the substantial underlying genetic variability. Differences in bacterial mutation rates act as a key evolutionary force, promoting adaptation and survival under high-stress environmental conditions.^25^ Hypermutator phenotypes emerge from initial silencing mutations in DNA replication/repair and oxidative stress mechanisms that enable the acquisition of advantageous/deleterious mutations at an accelerated rate.^26,27^ Environmental epigenetic stimuli facilitate the emergence and conservation of hypermutator lineages within bacterial populations.^28^ Mutations in the bacterial methyl-directed mismatch repair (MMR) system have been shown to enhance horizontal gene transfer between species.^29^ MMR-associated mutational patterns demonstrate increased C > T and *T* > C transition mutations, as well as frameshift mutations caused by insertions or deletions.^30–32^ In clinical environments, hypermutator *Pseudomonas aeruginosa* phenotypes promote host adaptation and rapid drug resistance.^33^ Studies have shown that hypermutator lineages can stabilize and further diversify across macroevolutionary time scales once adapted to their new environment.^27,34,35^ Mutations resulting in gene loss can reduce growth rates compared to wild-type strains; however, niche specialization may select for traits that reduce host recognition during infection or enhance competition in polymicrobial environments.^33,36^

Rhamnolipids (RL) produced by *P. aeruginosa* exhibit diverse properties such as surface tension reduction, emulsification, bioremediation, and antimicrobial effects, making them attractive green alternatives to conventional surfactants.^37–43^ The RL biosynthesis pathway is genomically distributed between two operons (*rhlAB* and *rhlC-PA1131*). RhlA converts β-hydroxyacyl-ACP to 3-(3-hydroxyalkanoyloxy) alkanoic acids (HAAs). RhlB catalyzes the first rhamnosylation reaction, in which dTDP-l-rhamnose is linked to HAAs through an α-glycosidic bond forming monorhamnolipids (M-RL) (Figure S1).^44,45^ RhlC catalyzes a second rhamnosylation reaction, producing dirhamnolipids (Di-RL).^46^ The function of the *PA1131*-encoding protein is still unclear.

HAAs are composed of two esterified β-hydroxy fatty acids with the length of their carbon chains (C8–C16) determining the biosurfactant’s physical properties such as micellization and emulsification.^47,48^ In *P. aeruginosa*, M-RL and Di-RL can be single- or double-tailed, while RL derived from *Burkholderia* display double-tailed congeners. In most cases, the fatty acid chains are saturated, but they can also be mono- or polyunsaturated. In double-tailed congeners, the fatty acid chains are linked via an ester bond between the β-hydroxyl groups of the distal chain and the carboxyl group of the proximal chain.^42,43^

RhlB is central to the RL biosynthesis. Its transferase activity and multidomain nature highlighted potential complexities in the biosynthetic process that would have been overlooked if the predominant focus resided with RhlA. A semirational approach was developed to capture naturally occurring distal mutations and their synergistic effect that conventional mutagenesis approaches might miss.^49^ Here, we established a multidomain DNA–protein mining pipeline to harness large sequence databases, identify functionally important segments, and provide comprehensive insights into enzyme function and substrate interactions that may enhance RL productivity. Three *rhlB* variants (*rhlB1*, *rhlB2*, and *rhlB3*) were identified through the mining pipeline, encompassing 38 amino acid and 178 nucleotide substitutions collectively observed across the variants. These constructs were experimentally validated using a modular *rhlAB* genetic construct expressed in *Escherichia coli*. A defined synthetic medium and high-throughput screening pipeline were developed to quantify monorhamnolipid production. This study proves the concept of utilizing computational methods to accelerate enzyme engineering, potentially informing the optimization of industrial bioproduction processes.

## Results and Discussion

### Developing a DNA–Protein Mining Pipeline

The mining pipeline integrated DNA and protein-level analyses. Mutations at the DNA level can influence transcription, translation, protein folding, stability, and activity.^50,51^ Missense mutations within active domains can alter enzymatic activity, while silent mutations can lead to a decrease in translation rate and affect mRNA half-life.^52,53^ Nucleotide changes may also introduce premature stop codons leading to truncated proteins.^54^ To characterize the mutational landscape of *rhlB*, a multilayer framework incorporating sequence analysis parameters at both DNA and protein levels was established using a repository of 8,096 sequences (Figure 1).

**Figure 1:**
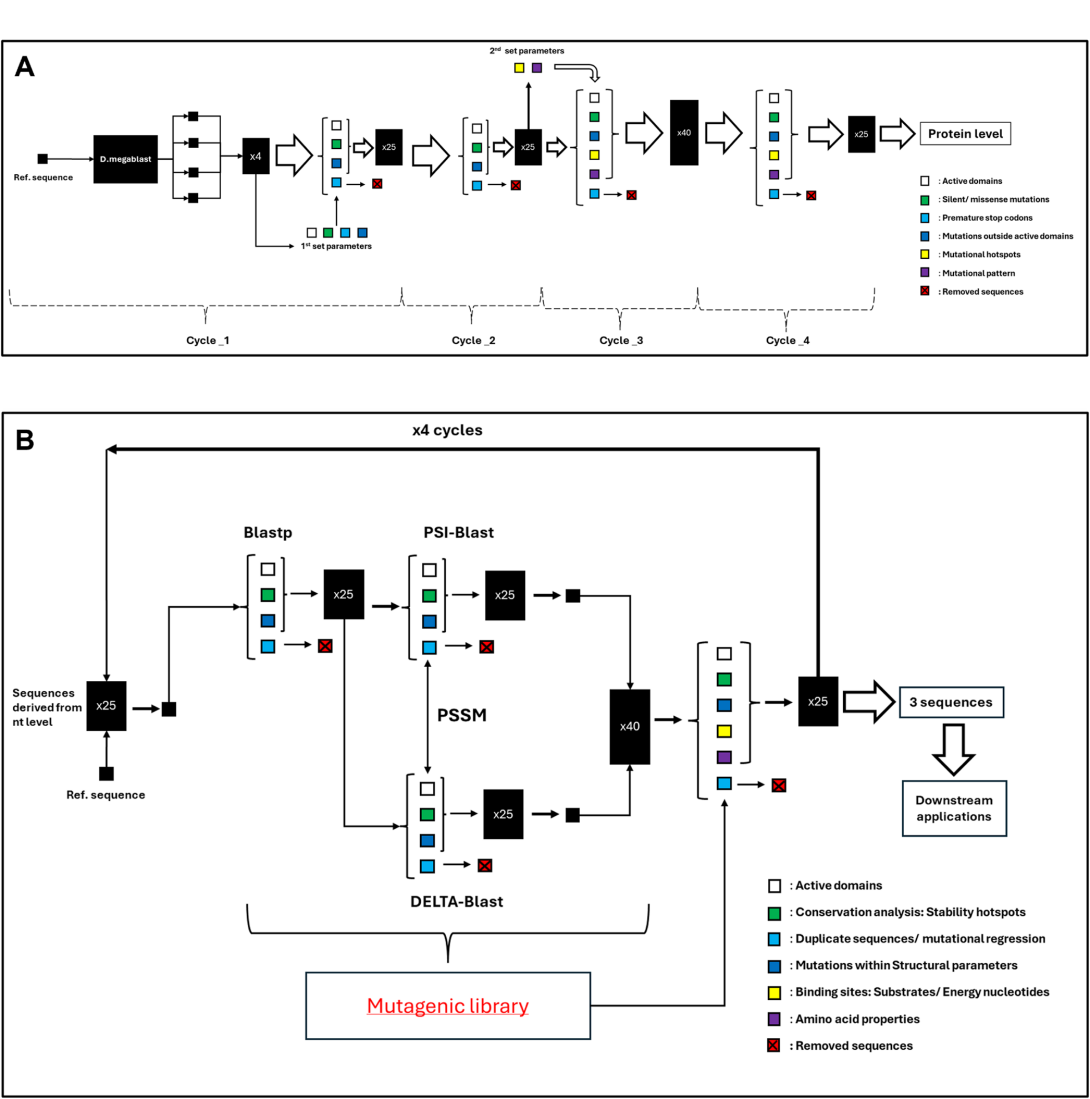
Schematic diagrams illustrating mining pipelines. (A) Nucleotide-Level Pipeline: A layered design is initiated with a discontinuous megablast search identifying four suitable entry sequences. Subsequent cycles analyze mutations within functional elements (active domains, envelope), classifying them by type (silent/missense, premature stop codons). Later cycles incorporate mutational hotspots and patterns. Each cycle utilizes defined hyperparameters (color-coded; see legend) with depths of ×25 (cycles 1, 2, and 4) and ×40 (cycle 3). The pipeline culminates in the selection of 25 sequences for protein-level analysis. (B) Protein-Level Pipeline: Inputting the 25 nucleotide-selected sequences, each cycle begins with a blastp search (250 seq) followed by PSI- and Delta-blast algorithms to construct a Position-Specific Scoring Matrix (PSSM). Analysis parameters focus on mutations within active domains, stability hotspots, structural elements (α-helices, β-sheets, turns, loops), conservation analysis, and substrate and energy nucleotide binding sites. Sequences exhibiting mutational regression or duplication are removed. Arrows demonstrate direction and pipeline information flow.

Given the sequence depth (<10,000), a four-cycle mutagenesis benchmark, common in enzyme engineering workflows with similar data set sizes,^55^ was selected for this study. The DNA mining pipeline began with the selection of a reference strain and genes intended for optimization. The first *P. aeruginosa* PAO1 genome to be sequenced was selected as the reference strain with its *rhlAB* serving as the reference sequences.^56^ A sequence repository was established by compiling genomic libraries (NCBI, *Pseudomonas* Genome Database) and integrating data on gene regulation, genomic location, GC/AT content, active domains (nucleotide/protein level), and structural information from protein databases (UniProt, EMBL-EBI, NCBI). To avoid bias toward the reference sequence, an initial discontinuous Mega-BLAST analysis was performed at the species level, generating a diverse alignment of sequences with varying similarity (95–99.2%). Four representative sequences, selected based on the similarity of active domains (domains A and B) to the reference sequence (high: 99%, medium: 97–98%, low: 95%), were used to initiate the mining process. Each cycle generated 250 sequences, incorporating multiple entry sequences identified and analyzed from previous cycles; cycle depths varied (25 entries for cycles 1, 2, and 4; 40 for cycle 3) to increase search breadth. Following each cycle, sequences were analyzed and categorized based on polymorphic variation patterns, location, and function. The repository was further refined by removing highly similar (>99%) or dissimilar (<75%) sequences, as well as those containing premature stop codons (TAA, TGA, TAG). A detailed step-by-step process is outlined in Figure S2.

Following data cleansing, sequences were analyzed first for AT/GC content and nucleotide counts (A, T, G, C). A subsequent analysis quantified total and per-line (60 nt) mutation percentages. After the second cycle, mutational pattern and hotspot analyses were introduced, leveraging a profile of *rhlB* mutations generated from the initial cycles. Pairwise DNA alignment identified conserved mutation regions with increased sequence entries in the third cycle (40 vs 25) designed to enhance variability for these targeted parameters (Figure 1). Sequences were categorized into groups based on mutation position within defined regions: active domain, envelope, and nonactive/envelope (Figure S2). This distribution informed the selection of new search entries representing specific or combined groups.

Mutations were classified as silent or missense; shared silent mutations served as lineage-specific anchors for further categorization. A nucleotide polymorphic motif was designated as a mutational pattern if present in >4 sequences (either at the same location or across multiple lines). A mismatch pattern matrix analyzed mutation distribution, subdividing patterns into consecutive and separated types. Shared pattern positions were used to refine sequence classification, while a scoring matrix compared mismatches per line against the reference sequence. Identified segments were aligned using the Needleman–Wunsch algorithm utilizing a custom substitution matrix. Nucleotide mutation patterns can serve as evolutionary traces, illuminating bacterial lineage divergence and subpopulation emergence.^57,58^ Analyzing these patterns also helped elucidate the RL pathway’s evolution, given that sequences originated from both clinical and environmental isolates. However, a lack of isolate origin data proved to be a significant limitation when using mutational patterns for accurate sequence categorization.

Comparing GC% across *rhlB* sequences revealed consistent values between 66% and 69%, with no major outliers relative to the reference. Mutations in *rhlB* predominantly favored G/C bases (70%), with A mutations being the least frequent (5%). T mutations increased alongside greater polymorphic variability and were particularly prevalent in sequences exhibiting lower GC content than the reference (67.9%), which displayed high identity percentages (97–98.9%) and a low overall mutation count (<12). For instance, *rhlB*1 showed 98.19% homology with the reference, featuring 10 nucleotide mutations (50% T substitutions), two of which resulted in amino acid changes. This sequence was selected for biological testing due to its T-mutation preference and location of substitutions within protein stability hotspots (Figure S3).

The subsequent candidate sequences (*rhlB*2 and *rhlB*3) favored G/C transversions, potentially indicating recombination events.^59^ In both sequences, mutations displayed sporadic patterns such as **G**C**G**, **G**CC**G**, and **G**GC**C**, with consecutive As replaced by Gs or Cs, with two-nucleotide mismatches separated by one or two bases. *rhlB*3 extended these patterns further, incorporating distances of two to four nucleotides between G/C mutations (e.g., **C**CG**C**GG**C**, **C**CCGG**C**CGG**C, G**GC**GC**). The observed mutational pattern displayed a high frequency of G/C substitutions replacing As, suggesting multiple mutagenic cycles aimed at stabilizing protein folding and function. *rhlB*2 contained 48 nucleotide mismatches (11 resulting in amino acid substitutions, 37 silent), while *rhlB*3 demonstrated 120 mutations (34 mismatches corresponding to 26 substitutions, 85 silent) (Figure S4).

Following DNA-level mining, 25 sequences progressed to protein-level analysis, requiring structural characterization of substrate/product binding sites, energy nucleotide binding motifs, and stability hotspots. Polymorphic variations were mapped onto the RhlB tertiary structure and classified according to active domains (domain A or B) and secondary structural elements (α-helices, β-sheets, loops, and turns) (Figure 2). This structural stratification enabled systematic categorization of substitutions by position and context, facilitating the identification of variants potentially influencing folding, stability, or conformational dynamics.

**Figure 2:**
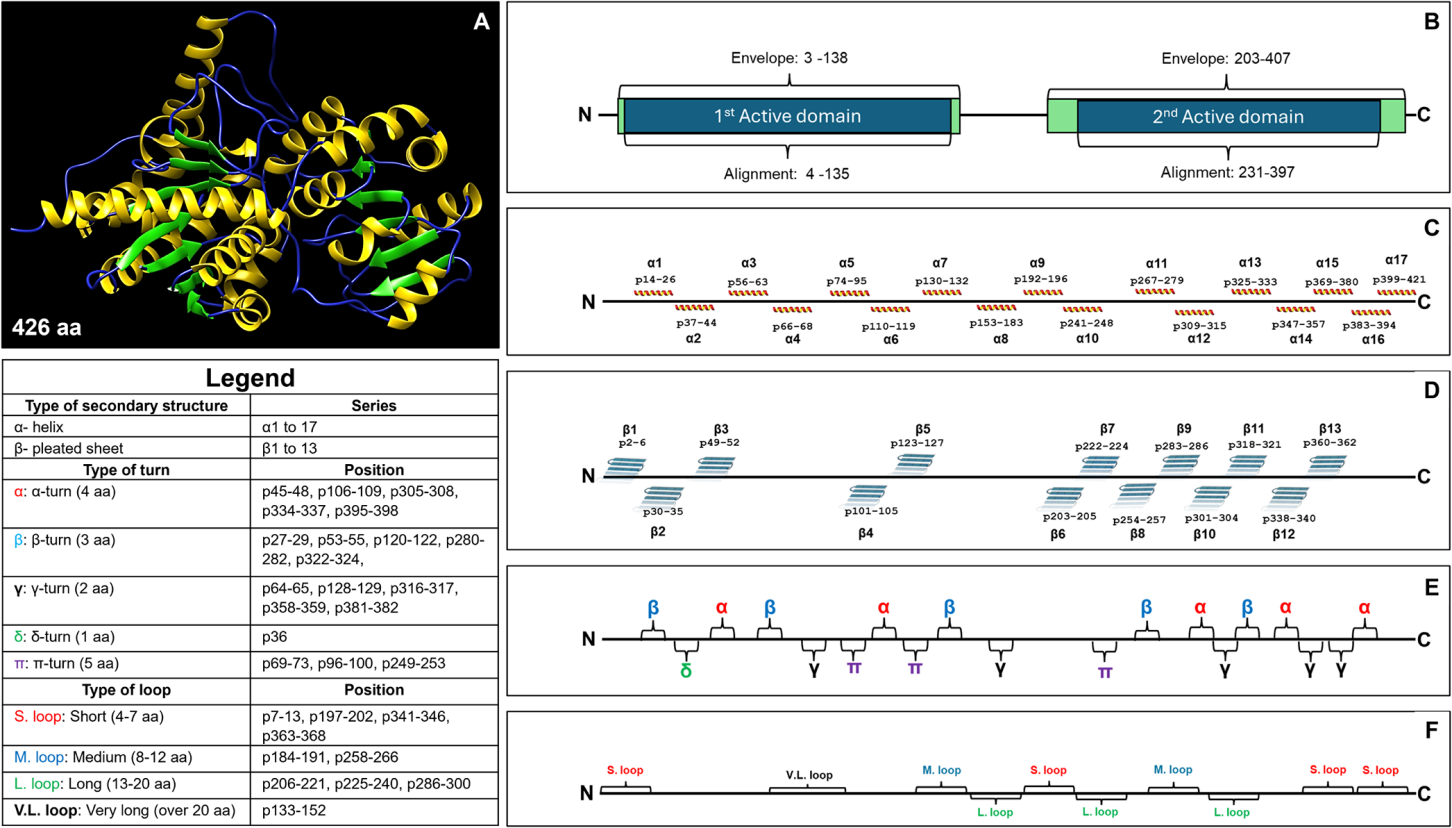
Structural analysis of RhlB. (A) Reference RhlB protein model (426 amino acids). (B) Localization of active domains within the RhlB sequence. (C, D) Distribution of α-helices and β-pleated sheets, showing amino acid string composition and sequence position. (E, F) Categorization of primary RhlB structure into turns and loops based on amino acid string length (see Legend).

### Substrate and Product Binding Sites in RhlB

To identify substrate and product binding sites, small-molecule docking simulations were performed using the reference RhlB, mapping potential binding sites for use in the mining pipeline. Sequences with mutations directly within these binding sites were omitted due to the predicted enzyme dysfunctionality. The focus was on identifying mutations near binding sites, aiming to enhance the substrate binding efficiency through increased site plasticity. Two docking simulations identified potential product binding sites (Figure 3). The first M-RL/RhlB simulation (AC score: −32.05, SwissParam score: −7.4) revealed four key residues: p396 (two hydrogen bonds with ligand, one with structure), p333 (one hydrophobic bond with ligand and structure), p22 (one ionic bond with ligand), and p230 (one hydrophobic bond with ligand). The second simulation (AC score: −30.6, SwissParam score: −9.034) identified seven additional sites: p11, p15, p260, p286, p306, p309, and p326, all interacting with the M-RL ligand. Both primary and secondary product binding sites were included for illustration purposes and to demonstrate site overlap with the substrate ligands (Figure 3A,B,G).

**Figure 3:**
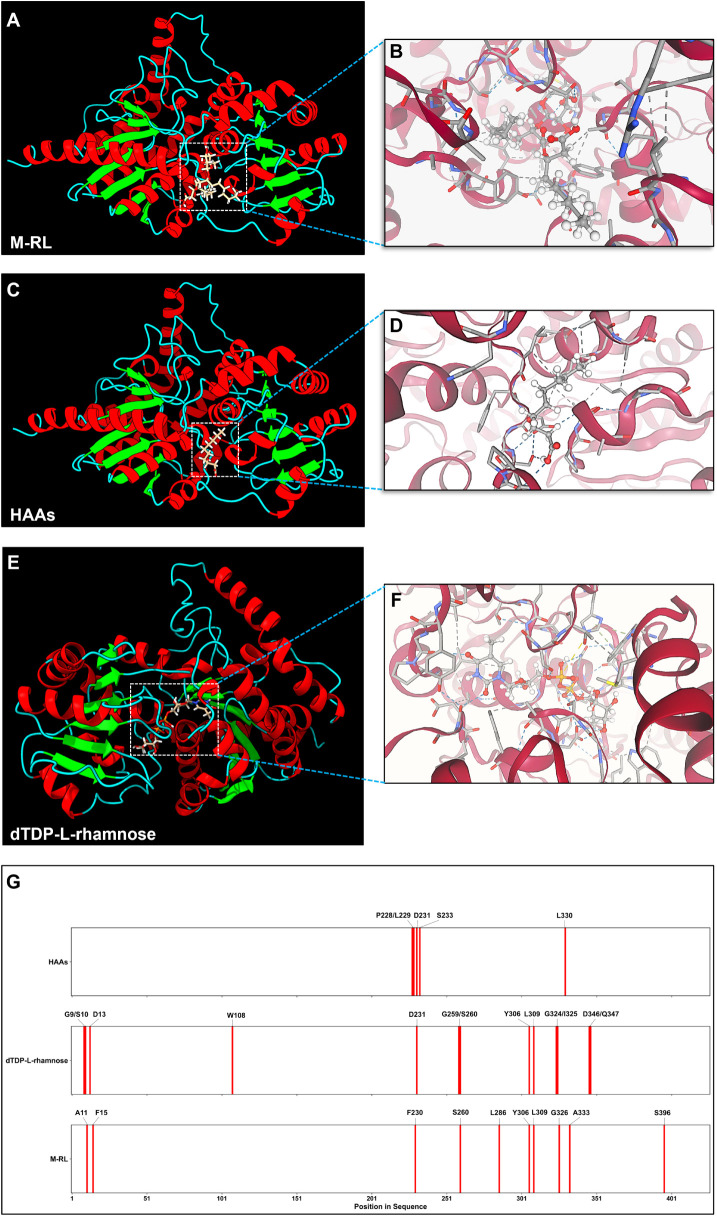
Small-molecule docking simulations within the reference RhlB. (A) Position of M-RL within the protein structure. (B) Intermolecular forces between M-RL and the protein. (C) Position of HAAs within the protein structure. (D) Intermolecular forces between HAAs and the protein. (E) Position of dTDP-l-rhamnose within the protein structure. (F) Intermolecular forces between dTDP-l-rhamnose ligand and protein. (G) Heatmap of substrate (dTDP-l-rhamnose and HAAs) and product (M-RL) binding regions mapped onto the RhlB reference sequence, with corresponding amino acid residues and positions indicated.

To understand how RhlB binds its substrates, we investigated both sugar and fatty acid moiety binding sites, with particular focus on HAAs as it had been previously identified as a limiting factor in M-RL biosynthesis.^60^ HAAs interact through two main segments: a large loop (p225–240) where residues p228, p229, p231, and p233 each form multiple hydrogen bonds with the carboxyl groups of the ligand; and a segment of the 13th α-helicase (p325–333), with residue p330 forming one hydrogen bond (AC score: −66.36, SwissParam score: −6.8; Figure 3C,D,G). Interestingly, adjacent residues F230 and I234 within the loop exhibited increased hydropathy. To accurately model these interactions, simulations utilized a C-10 HAAs molecule, reflecting the prevalence of C-10 fatty acid chains in common RL congeners.^44^ Simultaneously, the dTDP-l-rhamnose binding site was distributed mainly across loops, α and β turns, and α-helicases (AC score: −314.62, SwissParam score: −9.81). In domain A, only the short loop (p7–13) displayed three amino acids (p9, p10, and p13) forming hydrogen bonds with the ligand, with p13 also forming an ionic bond. The majority of binding sites were identified in domain B, consisting of the long (p225–240) and medium (p258–266) loops demonstrating three binding residues with p231 forming a hydrophobic bond and p259 and p260 forming 1 and 3 hydrogen bonds with the ligand, respectively. The α-turn (p305–308) displayed one binding site at p306 and the adjoining 12th α-helix (p309–315) at p309, both contact points formed one hydrophobic bond with the ligand. Following a similar distribution pattern, the β-turn (p322–324) displayed one residue at p324 and the adjoining 13th α-helix (p325–333) displayed one residue at p325, both sites forming single hydrogen bonds. The short loop (p341–346) and adjacent 14th α-helix (p347–357) displayed two contact points at p346 and p347, with each forming a single hydrogen bond (Figure 3E–G). Both substrates shared multiple binding sites with M-RL (Figure 3G). Given substrate position and proximity analysis, the catalytic site was hypothesized to reside within domain B near the dTDP-l-rhamnose ligand; therefore, M-RL binding sites were excluded as selection parameters for candidate sequences. This further reinforced active site localization within the RhlB tertiary structure and computational biomolecular cavity analysis (Figure S5).

To analyze sequence variations, we created a scoring matrix identifying key positions within the reference sequence, focusing on substitutions at both binding sites (dTDP-l-rhamnose and HAAs) and adjacent residues (within a three-amino-acid distance). Sequences with mutations at the defined binding sites were excluded as being potentially dysfunctional. Amino acid properties (hydropathy, charge, and polarity) were used to further categorize the polymorphic variations identified. Interestingly, RhlB3 exhibited mutations in positions adjacent to both substrate binding sites.

To investigate whether RhlB requires energy nucleotides for activity, we analyzed potential binding sites using nucleotide motif searches and small-molecule docking simulations. Both ATP and GTP were identified as likely ligands (Table S6); docking simulations confirmed ligand interaction in the same vicinity (Figure S6). Motif search revealed 17 hotspots for ATP and 7 for GTP; docking simulations revealed 11 for ATP and 7 for GTP (Figure 4A). There was no similarity between the GTP docking and motif search results, while ATP shared 6 hotspots. Both ATP and GTP demonstrated similar distribution across both active domains, with domain B containing the majority of binding sites. Interestingly, both ATP and dTDP-l-rhamnose shared a number of docking contact residues (10, 13, 260, 306, 346, and 347). To increase parameter accuracy, binding sites were separated into two distinct groups: predicted (motif search) and docking. Proximity analysis of RhlB sequences revealed patterns of mutation distribution within or near to ATP/GTP sites (Figure 4B,C), suggesting a potential role for energy nucleotides in enzyme function. To determine if RhlB requires activation energy, RhlB2 and RhlB3 were selected for downstream processing as they both displayed mutations either within or adjacent to ATP/GTP binding sites.

**Figure 4:**
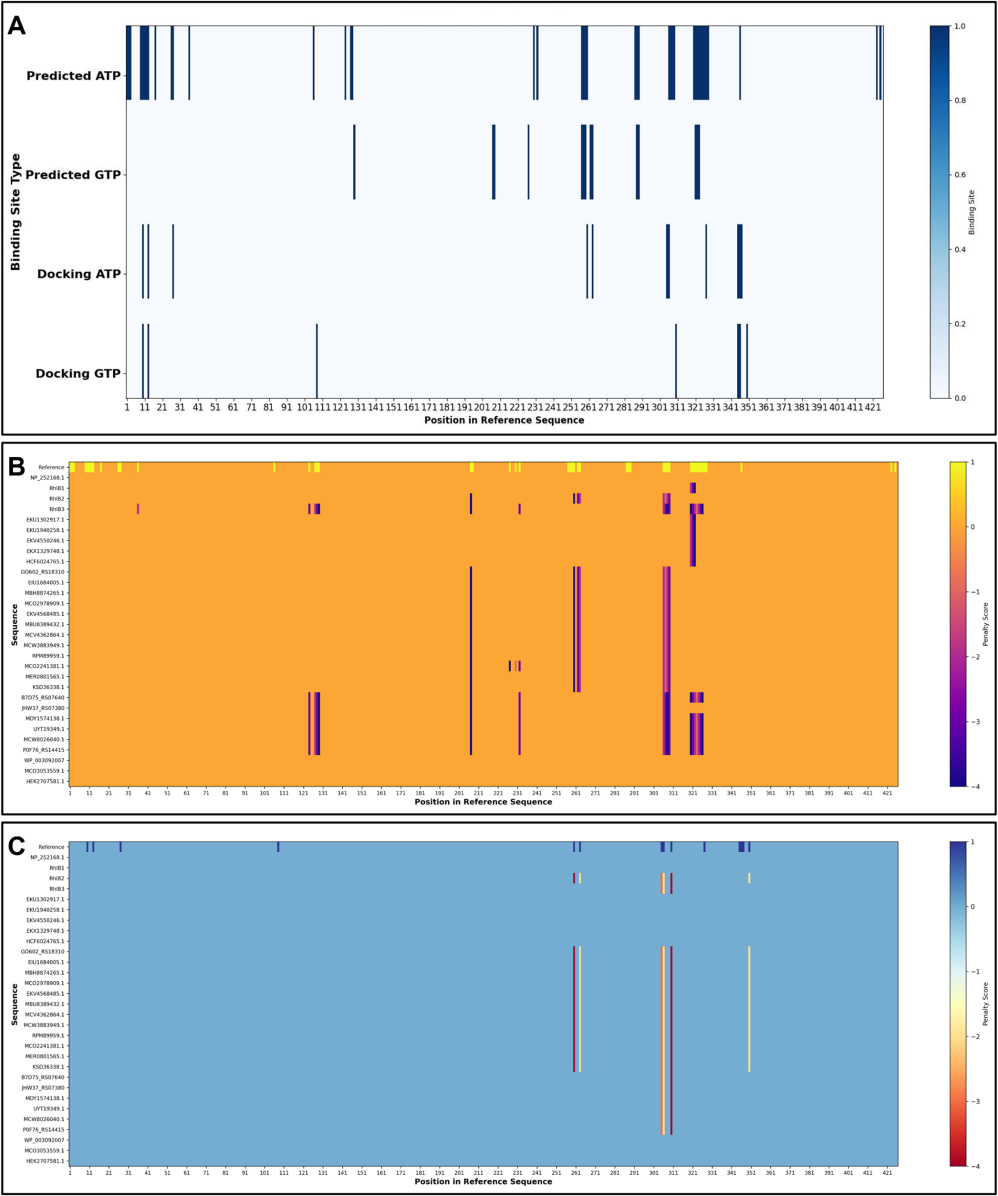
Comparison of predicted and docking-simulated ATP/GTP binding sites. (A) Heatmap comparing predicted and docking sites within the reference RhlB sequence; blue indicates binding site positions, with color intensity reflecting amino acid cluster density. (B, C) Mismatch proximity heatmaps relative to predicted (B) and docking (C) binding sites. Sequences used for these heatmaps were selected for phylogenetic analysis of RhlB candidates, as several anchor mutations (e.g., p263, p305, p307), identified as ATP binding sites, served as common evolutionary markers. A total of 30 sequences, including the reference RhlB, were analyzed. Heatmap legends indicate proximity to binding sites (+1 position in reference sequence, 0 no mismatch, −1 same position, −2 to −4 amino acid distances).

### Establishing Experimental Framework

A modular *rhlAB* genetic construct (Figure S7) and a high-throughput colorimetric assay were established to quantify M-RL production from *E. coli* mutants expressing *rhlB* variants identified through the DNA–protein mining pipeline. The Victoria Blue (VB) assay, adapted from Kubicki et al.,^61^ was optimized for a 96-well format to enable quantitative detection of M-RL in bacterial supernatant. A new standard curve was generated using the supernatant from an *E. coli* mutant carrying the empty pBAD24 vector cultured in the *E. coli* Screening Medium (ESM), ensuring that background exoproducts were included. The VB detection range was 10–170 μg mL^–1^, with a limit of detection (LOD) of 8.8 μg mL^–1^and a mean absolute percentage error (MAPE) of 0.75% (Figure 5A).

**Figure 5:**
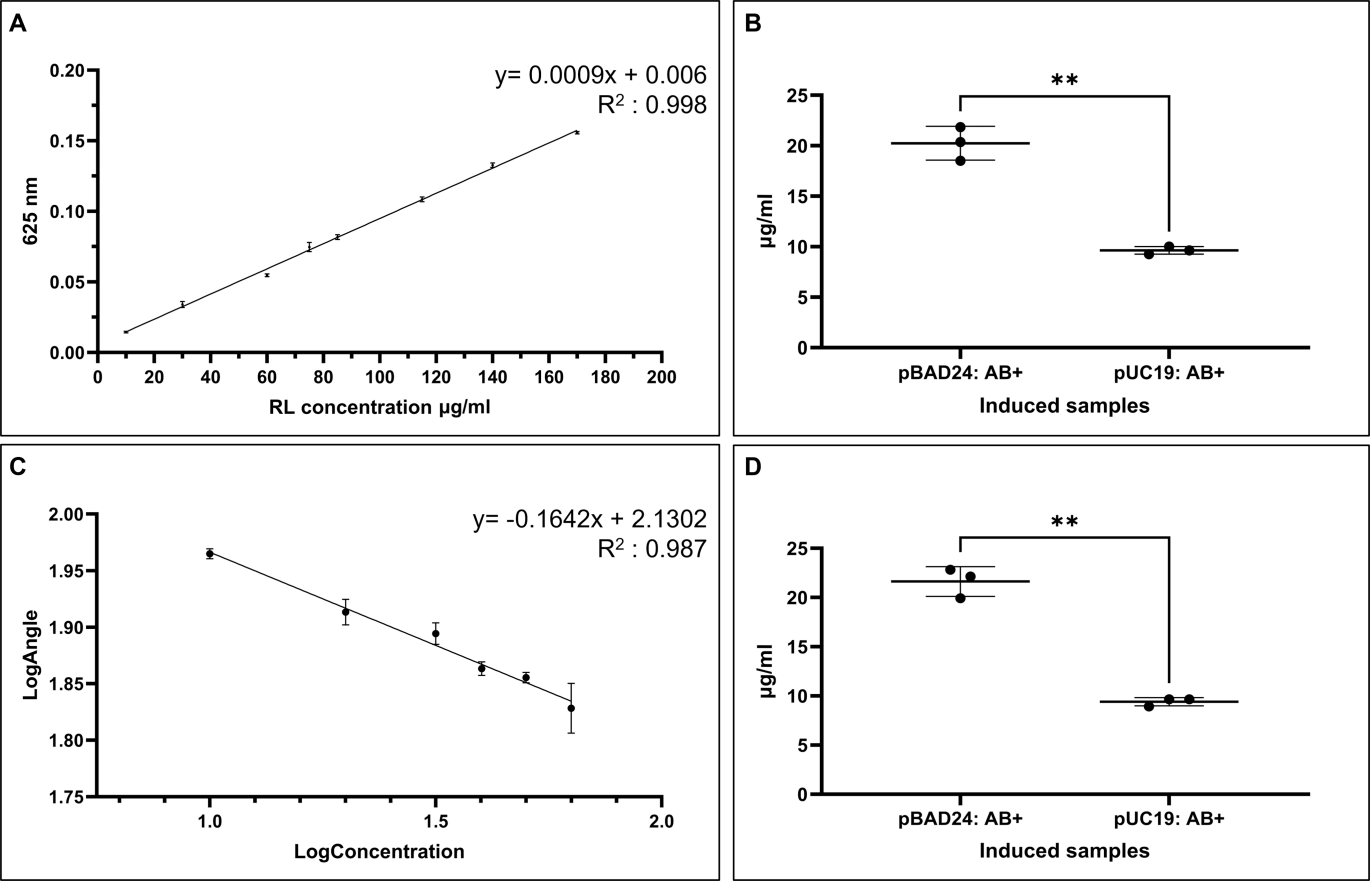
Reference constructs (pUC19-*rhlAB* and pBAD24-*rhlAB*) for M-RL production threshold and quantification assay standards. (A) Victoria Blue *E. coli* Screening Medium (ESM) DH5α supernatant RL standard. Cell-free supernatant was used to create the standard using commercial RL at concentrations ranging from 10, 30, 60, 75, 85, 115, 140, and 170 μg mL^–1^. (B) Quantification of M-RL production of Victoria Blue. The concentration for pBAD24-*rhlAB* was 20.1 μg mL^–1^ and for pUC19-*rhlAB* 9.56 μg mL^–1^. Samples were analyzed using Welch’s *t* test (two-tailed). (C) Rhamnolipid contact angle standard. Commercial RL standard in ESM, presented on a logarithmic scale in brackets. The concentrations of RL ranged from 10 (1), 20 (1.3), 30 (1.5), 40 (1.6), 50 (1.7), and 60 (1.8) μg mL^–1^. (D) Quantification of M-RL using the contact angle assay. The mean concentration for pBAD24-*rhlAB* was 21.63 μg mL^–1^ and for pUC19-*rhlAB* 9.41 μg mL^–1^. Samples were analyzed using Welch’s *t* test (two-tailed). All samples were grown in ESM, with pUC19-*rhlAB* induced by 0.4 mM IPTG and pBAD24-*rhlAB* by 0.7% arabinose, respectively. Induced samples are indicated by a “+”. Victoria Blue absorbance: 625 nm. Statistical significance: **p = 0.0021.

To verify assay accuracy, a contact angle method based on the surface-tension-reducing property of rhamnolipids was established using commercial RL droplets (8 μL) on hydrophobic PDMS surfaces (Figures 5C and S8). A linear correlation between RL concentration and contact angle was observed within 0–100 μg mL^–1^ (MAPE 2.67%), aligning with the VB assay range. Together, these results confirmed that the VB assay could quantify the M-RL concentration in bacterial supernatants.

Expression levels of the reference *rhlAB* construct were then evaluated by using both pUC19 and pBAD24 vectors (Figure 5B). A range of arabinose concentrations (0–1%; w/v) for induction was tested, and 0.7% was identified as the optimal concentration for pBAD24 induction, as higher levels caused system saturation and reduced M-RL yield (Figure S9). M-RL production output from pBAD24-*rhlAB* was at 20.1 μg mL^–1^, significantly higher than 9.56 μg mL^–1^ from pUC19-*rhlAB* (*p* < 0.0061). Contact angle assays gave comparable values as 21.63 μg mL^–1^ and 9.41 μg mL^–1^, respectively, with a significant difference (*p* < 0.003) (Figure 5D). Minor discrepancies between the assays reflected the measurement principles (solubilization vs wettability) but confirmed the robustness of the VB quantification method.

Using this validated assay framework, media optimization was performed to support rhamnolipid biosynthesis and ensure compatibility with both VB and oil diffusion assays. The initial use of Luria–Bertani (LB) medium produced false positives (Figure S10), confirmed by oil diffusion analysis (Tables S1 and S2), indicating interference from LB components. Consequently, a defined induction medium was formulated and systematically optimized.

Individual LB components (yeast extract, peptone, NaCl) were evaluated using an M9 minimal medium base with different carbon (glucose, sucrose, l-rhamnose) and amino acid sources (casamino acids, tryptone), while maintaining glycerol as a fatty acid precursor for HAAs biosynthesis (Table S10).^62^ Using *E. coli* pUC19-*rhlAB* as a reference, the glucose-based medium (media 6) yielded the highest M-RL production (*p* < 0.045) when compared to sucrose or l-rhamnose (Figure 6B). Casamino acids significantly enhanced production relative to tryptone (*p* < 0.0073), whereas peptone (media 4) interfered with the VB assay by displacing the dye, consistent with the oil diffusion results (Figure 6A–C).

**Figure 6:**
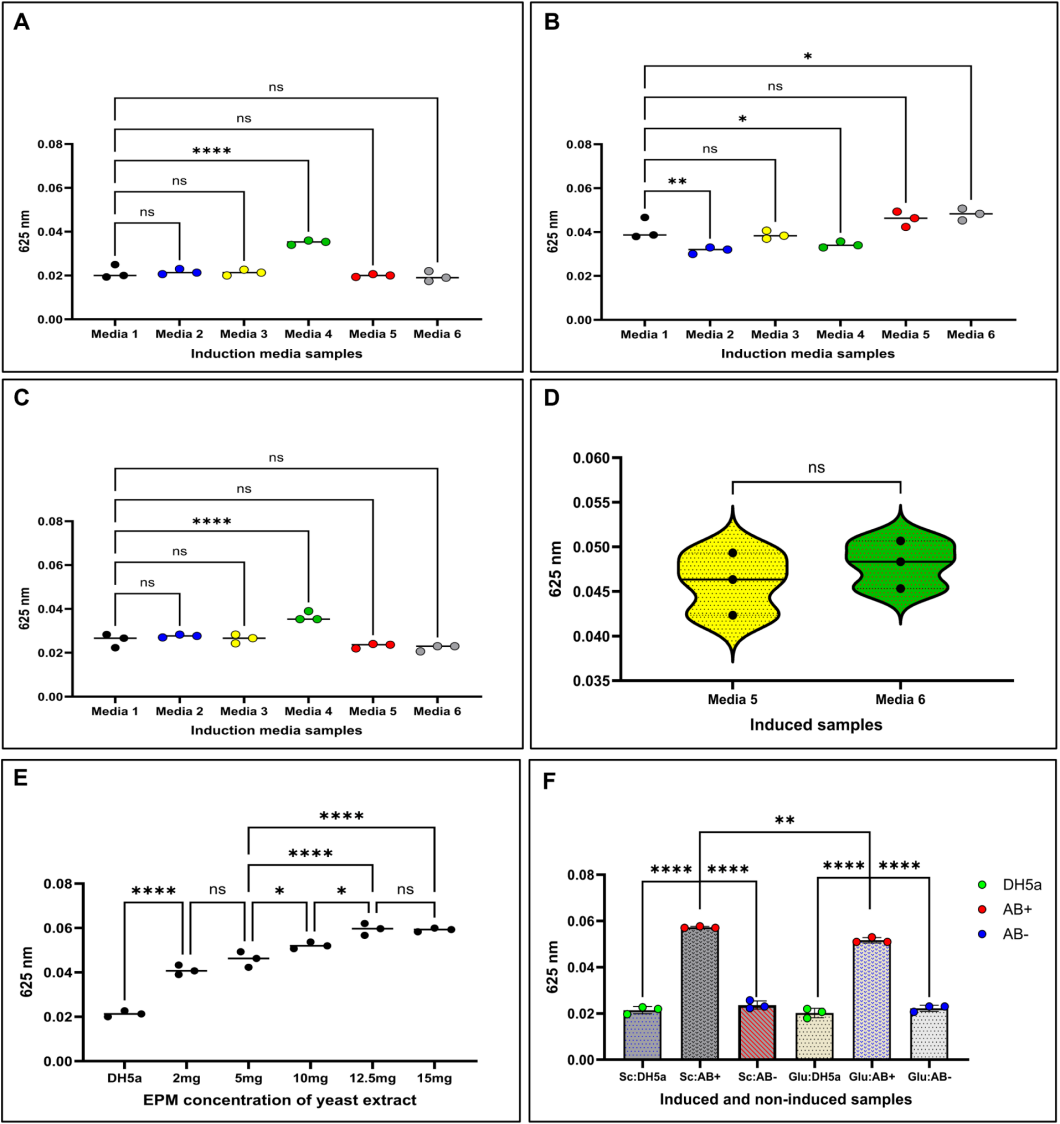
Optimisation of *E. coli* induction media for M-RL production and Victoria Blue assay compatibility. Six media formulations were tested (Media 1–6; Table S10). M-RL production was evaluated using the pUC19-*rhlAB* reference clone, with absorbance at 625 nm measured via the Victoria Blue dye assay. (A) DH5α control samples across different induction media. (B) Noninduced samples in respective media. (C) Comparison of induced M-RL production across all media. (D) Induced (AB+) M-RL production output comparison between Media 5 and Media 6. (E) Effect of yeast extract concentration (2, 5, 10, 12.5, 15 mg mL^–1^) on M-RL biosynthesis; DH5α served as a control. (F) Comparison of sucrose and glucose as carbon sources; controls included empty bacterial host DH5α (green), noninduced pUC19-*rhlAB* clone (blue), and induced pUC19-*rhlAB* clone (red). Induced (+), Noninduced (−), Sc: sucrose, Glu: glucose. Statistical significance: ns = 0.1234, *p = 0.0332, **p = 0.0021, ***p = 0.0002, ****p < 0.0001.

Sucrose with yeast extract achieved production comparable to that of glucose (Figure 6D). Further optimization identified 12.5 mg mL^–1^ yeast extract as optimal (*p* < 0.0147), with higher concentrations yielding no additional increase (Figure 6E). Under optimized conditions, sucrose slightly outperformed glucose (*p* < 0.0085) (Figure 6F). Although M9 salts caused minor oil diffusion artifacts prior to culturing, they did not affect the VB quantification. To eliminate residual interference, the final ESM replaced M9 salts with NaCl, retaining sucrose, yeast extract, glycerol, and casamino acids at optimized concentrations (Table S11). ESM supported consistent M-RL production and compatibility across all assays and was used for subsequent screening of *rhlB* polymorphic variants.

### Rhamnolipid Production from the RhlB Variants

To establish the evolutionary context of the observed functional differences, a Maximum Likelihood phylogenetic analysis was performed on 30 RhlB sequences, rooted with a glycosyltransferase sequence from the environmental *Pseudomonas shahriarae*.^63,64^ This analysis confirmed that the three candidate sequences (RhlB1, RhlB2, and RhlB3) represent distinct lineages that diverged from the PAO1 reference RhlB (Figure 7, Table S9).

**Figure 7:**
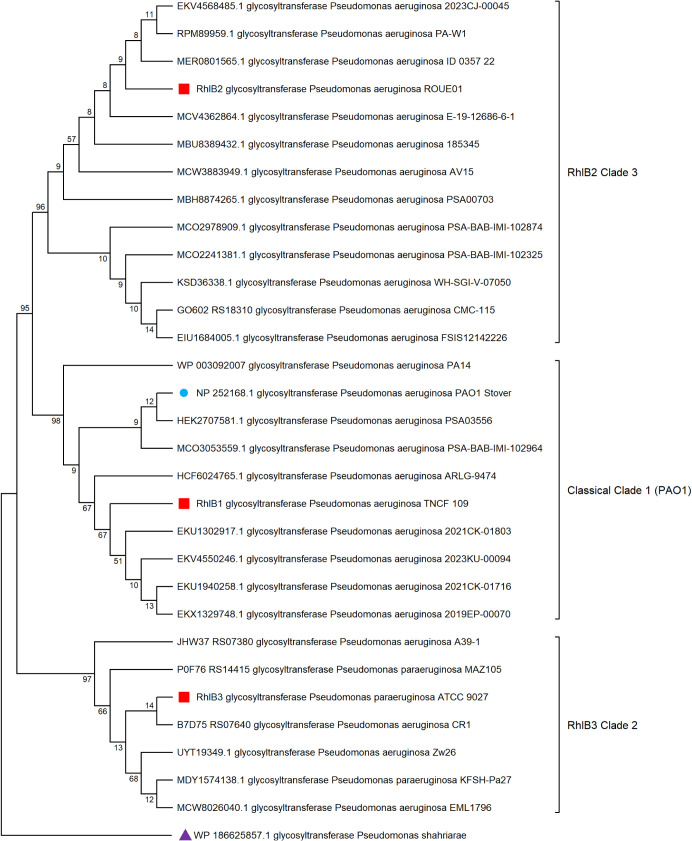
RhlB phylogenetic tree analysis. The evolutionary relationship between RhlB variants was calculated using the Maximum Likelihood method and JTT matrix model, incorporating 30 RhlB sequences (length: 426 amino acids). The PAO1 reference RhlB sequence is indicated by a blue circle, while the candidate sequences (RhlB1, RhlB2, and RhlB3) are represented by red squares. The phylogenetic tree was rooted using the outgroup glycosyltransferase sequence from *Pseudomonas shahriarae* illustrated by a purple triangle. Phylogenetic clades are represented by using brackets. Evolutionary divergence between sequences is shown next to each branch as the percentage of replicate trees in which the associated taxa clustered together (1,000 bootstrap replicates). Protein ID, protein type, and strain ID are provided.

The sequence analysis further categorized RhlB2 and RhlB3 as belonging to distinct hypermutator outlier phylogroups relative to the classical *P. aeruginosa* clade (Figure S11). Sequence conservation mirrored these evolutionary distances: *rhlB1* showed 98.19% nucleotide similarity (and 99.53% protein identity) to the reference, while divergent *rhlB3* showed the lowest similarity (90.01% nucleotide, 94.13% protein identity). The shared presence of five substitutions (p189, p204, p248, p299, and p307) between RhlB2 and RhlB3 indicated a common ancestral history despite their dramatic divergence in function.

Functional screening using the VB assay to quantify the M-RL production output revealed significant phenotypic differences (Figure 8). Normalized against the *E. coli* host background, the induced reference construct (iAB) produced 20.1 μg mL^–1^ of M-RL. The induced RhlB1 construct (iAB1) produced 22.18 μg mL^–1^, a minor, nonsignificant 1.1-fold increase. In contrast, the RhlB2 construct exhibited profound dysfunction, with production below the assay’s LOD when uninduced and approximately 3.2 μg mL^–1^ when induced, representing a 6.3-fold reduction. This suggested that RhlB2 was unable to catalyze the first rhamnosylation reaction, despite SDS-PAGE confirming its proper expression (Figure S12). Gene expression analysis further supported the SDS-PAGE results demonstrating no significant expression difference between *rhlB* variant constructs (Figure S13). The induced RhlB3 construct (iAB3) demonstrated the highest output, achieving 55.51 μg mL^–1^, which translated to a significant 2.76-fold enhancement (*p* < 0.0001) over the reference.

**Figure 8:**
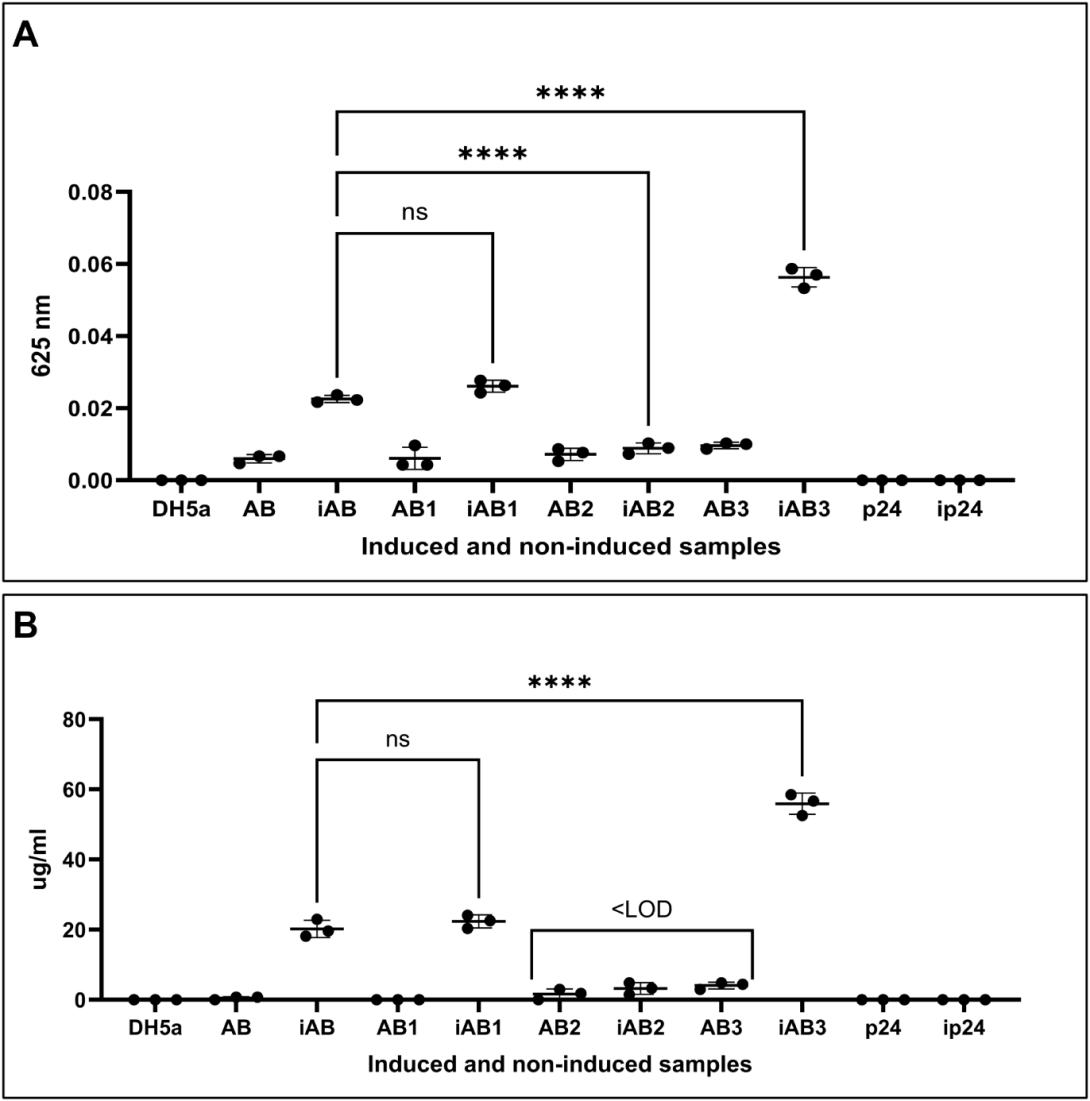
Comparison of M-RL production between*rhlB* variants using the Victoria Blue assay. (A) Absorbance readings of the *rhlB* variant constructs. Positive controls were the empty bacterial host DH5α and the uninduced empty vector pBAD24 (p24); the induced empty vector pBAD24 (ip24) served as the negative control. For each sample, both induced (i) and uninduced samples were tested. Background absorbance of ip24 was subtracted from all samples. Construct designations: reference pBAD24-*rhlAB* (AB), pBAD24-*rhlAB*1 (AB1), pBAD24-*rhlAB*2 (AB2), pBAD24-*rhlAB*3 (AB3). Samples were statistically analyzed using one-way ANOVA with Dunnett’s multiple comparison test. Victoria Blue dye absorbance was measured at 625 nm. (B) Corresponding M-RL production (μg mL^–1^) for each *rhlB* variant construct. M-RL production was quantified for each biological replicate using the equation *y* = 0.0009*x* + 0.006, derived from the ESM Victoria Blue RL standard. Concentrations below the Limit of Detection (LOD) of the standard (8.8 μg mL^–1^) are indicated as <LOD. Samples were statistically analyzed using one-way ANOVA with Dunnett’s multiple comparison test. Statistical differences between samples are denoted as ns = 0.1234, ****p < 0.0001.

### Sequence and Structural Interpretation of Functional Variation

Comparative molecular modeling and analysis (Figures 9 and S14–S21) were employed to interpret the structural mechanisms underlying the observed functional outcomes, focusing on how polymorphic variations affected key catalytic domains, energy binding sites, and overall protein compactness (κ value).

**Figure 9:**
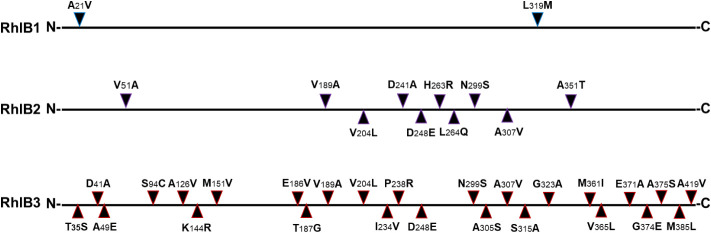
Position of polymorphisms in the RhlB sequences. The RhlB protein is 426 amino acids long, with the N-terminal (**N**) and C-terminal (**C**) domains indicated. Mutations are illustrated by arrows, showing the native amino acid from the reference RhlB sequence on the left and the corresponding amino acid substitution in each variant RhlB sequence on the right.

RhlB1 variant showed a minor enhancement in production. The two substitutions in RhlB1, A21V (in α1) and L319M (in β11), were analyzed for their impact. Both the fractional charge residues (FCR) and the κ value remained unchanged relative to the reference, suggesting a stable tertiary structure (Figure S21). Mechanistically, the A21V substitution created a conserved hydrophobic network with an adjacent β-sheet (p30–p35), altering hydrophobic cluster 1 (Figures S14 and S15). Similarly, the internal L319M substitution modified hydrophobic cluster 5. Both p21 and p319 reside in known hotspot regions for protein stability. Additionally, the p319 mutation was found in proximity (5 amino acids) to a dTDP-l-rhamnose and a predicted ATP (1 amino acid) binding site. These findings demonstrated that distal mutations within each of the protein’s active domains that restructure the hydrophobic network within α-helicase and β-sheet secondary structures can lead to a minor 1.1-fold increase in M-RL production.

RhlB2 contained 10 amino acid substitutions, sharing 3 mutations in active domain B and 2 in the interdomain region with RhlB3. These mutations were not responsible for the inactivity observed, as they would have also led to RhlB3 dysfunctionality. The near-complete loss of function in RhlB2 was traced to two unique substitutions (H263R and L264Q) located in a loop segment between β8 and α11, a region identified through molecular docking simulations as a putative ATP binding site. The overall κ value for RhlB2 was 0.15, compared to 0.17 for the reference RhlB (Figure S21), suggesting an increase in tertiary structure compactness. The H263R substitution replaced Histidine (a Brønsted acid/base) with Arginine (a polar cation). Histidine is recognized for its role as an efficient proton shuttle in enzymatic catalysis, contributing to catalytic efficiency through the unique properties of its imidazole side chain.^65^ Additionally, L264Q replaced nonpolar Leucine with polar Glutamine. This shift affected the polarity profile and profoundly restructured the local hydrogen-bond matrix, resulting in new stronger intermolecular bonds. These additional forces applied stronger compression to the loop, confirmed by dihedral angle analysis (Figure S19), which ultimately altered the geometry. Moreover, both H263R and L264Q were identified in close proximity to 3 and 4 amino acid distances to a dTDP-l-rhamnose binding site (p260; AC score: −312). These alterations suggested steric occlusion and a fundamental compromise of the conformational dynamics necessary for energy nucleotide binding at the ATP site, resulting in catalytic failure. This is further supported when AC docking scores are compared between the RhlB (−451.61) and RhlB2 (−444.82) models binding the ATP ligand.

The RhlB3 variant showed a significant 2.76-fold enhancement in M-RL productivity. RhlB3 contained a total of 26 substitutions with 12 mutations located in the primary structure, 9 in α-helices, and 5 mutations in β-sheets. The distribution of mutations in RhlB3 demonstrated a mosaic pattern with hotspot regions being identified at p186–189, p305–307, and p371–375. Additionally, a GTP binding site located on a β-turn PGG (p321–324) and an ATP binding site located on an α-turn AYAP (p305–308) were identified in close proximity to the HAAs binding site, as well as overlapping with dTDP-l-rhamnose binding sites. RhlB3 displayed two mutations, A305S and A307V, in the putative ATP site. The mutation at p305 affected the plasticity of the α-turn by lowering the hydrophobic interactions of native Alanine. Mutation A307V was shared with RhlB2, and in both protein models, Valine altered hydrophobic interactions within the fifth hydrophobic cluster, increasing structural compactness. It is hypothesized that ATP and GTP cause conformational changes allowing RhlB to interact with both the dTDP-l-rhamnose and HAAs substrates, facilitating the enzyme’s catalytic activity, with ATP/GTP utilization depending on the bacteria’s life cycle and availability.^66–68^

The increase in M-RL production was attributed to mutations targeting HAAs substrate affinity and membrane-based pathway efficiency. Two mutations (I234V and P238R) were identified in the HAAs binding domain loop (p225–240). The first mutation resulted in only a minor decrease in hydropathy. The substitution of P238R (nonpolar Proline to basic polar Arginine) increased the polarity and charge of this region. Dihedral angle analysis suggested a geometrical shift that correlated with a lower HAAs docking affinity score (RhlB3: −68.55 vs RhlB: −66.4) (Figure S20). This result suggests that the altered loop geometry enhanced the plasticity of the active site, leading to improved efficiency in binding the lipid substrate.^69^

A unique and crucial substitution, S94C, occurred in α5 (p74–p95). Cysteine at p94 restructured the local hydrogen network, forming new intermolecular interactions with p119 that connect α5 with the adjacent transmembrane anchoring domain α7 (p110–p199). This structural shift is proposed to optimize the geometric positioning of RhlB relative to RhlA at the membrane interface (Figure S18).^70^ Since membrane anchoring is known to drive enzyme–enzyme interactions by confining components to the 2D membrane plane^71,72^ the S94C mutation in RhlB3 may enhance the organization and thus the overall efficiency of the M-RL biosynthesis pathway, contributing significantly to the observed hyperproduction phenotype.^73–87^

### Essentiality of H263 for RhlB Catalytic Activity

To confirm that the H263R substitution was the primary cause of RhlB2 dysfunction and to validate the essentiality of H263 for RhlB catalytic activity, reverse mutation analysis was conducted. Reverting the H263R mutation back to the native Histidine (p263_A construct, R263H) successfully restored functionality, yielding 24.63 μg mL^–1^ M-RL production (a 1.22-fold increase over the reference, *p* < 0.032) (Figure 10). In contrast, the control construct R263T (p263_C construct) remained dysfunctional. Structurally, the R263H model’s κ value increased from 0.15 (RhlB2) to 0.16, indicating a decrease in tertiary structure compactness that would facilitate access to the energy nucleotide binding site. These findings demonstrate that H263 is essential for maintaining the catalytic function of RhlB. Our results suggest that mutations at this position disrupt key intermolecular interactions required for the stability of the active site and the binding of the dTDP-l-rhamnose substrate, thereby eliminating enzymatic activity.

**Figure 10:**
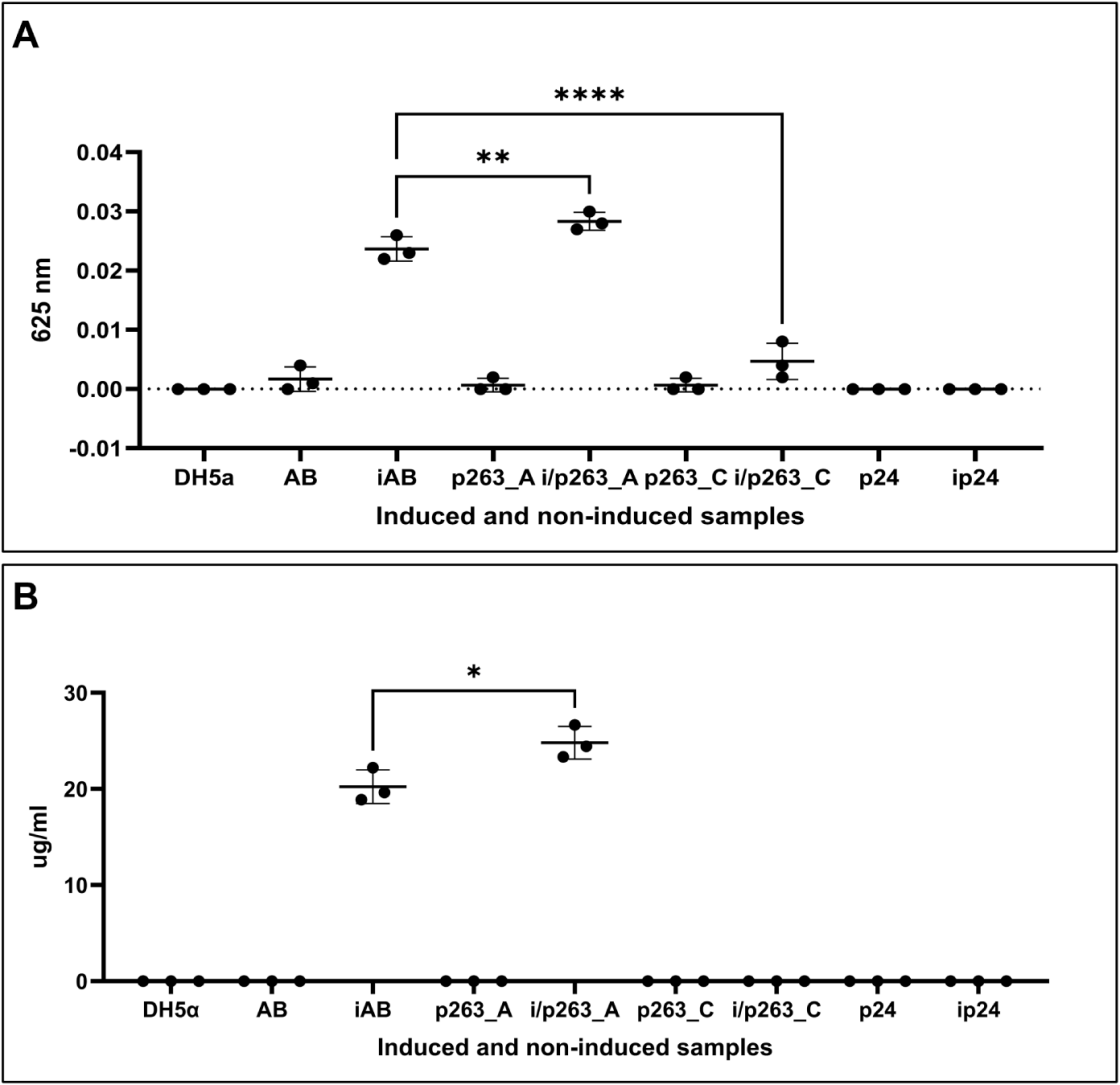
Second polymorphic variation cycle for reverting functionality in RhlB2. (A) Absorbance readings (625 nm) of the RhlB2-p263 constructs. Positive controls were the empty bacterial host DH5α and the uninduced pBAD24 vector (p24); the induced pBAD24 vector (ip24) served as the negative control. Both induced and uninduced samples were tested for each construct, with background absorbance (ip24) subtracted from all readings. Constructs: p263_A (Arginine substituted by Histidine), p263_C (Arginine substituted by Threonine). Statistical analysis was performed using one-way ANOVA with Sidak’s multiple comparison test. The p263_C construct served as a site-directed mutagenesis control, as Threonine’s proton acceptor properties result in neutral or negative charge. (B) M-RL production (μg/mL) for RhlB2-p263 constructs. Production was quantified using the equation *y* = 0.0009*x* + 0.006 derived from the ESM Victoria Blue RL standard. Statistical analysis was performed using a two-tailed Welch’s *t* test. Significance: *p = 0.0332, **p = 0.0021, ****p < 0.0001.

### Integrative Evolutionary Implications

Phylogenetic analysis of the RhlB variants demonstrates a rapid and divergent evolution of the M-RL biosynthesis pathway, driven by distinct ecological selective pressures. Phylogenetic and functional analyses identified two RhlB outlier phylogroups (RhlB2 and RhlB3) with opposing M-RL activity, providing a framework for interpreting the structure–function relationship and evolutionary plasticity of this key enzyme. The data suggested an ecological segregation with RhlB2 variants enriched in clinical pulmonary isolates demonstrating reverse zoonosis capabilities, whereas RhlB3 variants were more frequently associated with environmental or acute infection strains. A detailed epidemiological and evolutionary analysis of these variants is provided in the Supplementary Information for Discussion.

## Conclusion

This study investigated how polymorphic variations enhance M-RL production by analyzing RhlB sequences. Experimental validation revealed 178 nucleotide mutations and 38 amino acid substitutions across three RhlB variants, with the RhlB3 construct exhibiting the highest M-RL output (55.51 μg mL^–1^). RhlB3 contained a high density of mutations (26 total), impacting both loop/turn regions and secondary structures (β-sheets and α-helices), leading to altered hydropathy, polarity, and optimized protein packing.^88,89^ Interestingly, a mutation in the transmembrane domain (S94C) likely influences membrane localization and protein interactions through cysteine modification and potential palmitoylation.^90–92^ Mutations adjacent to the HAAs binding site (I234V and P238R), supported by molecular docking simulations, are believed to enhance substrate binding affinity via increased active site loop plasticity.^93^ Collectively, these findings demonstrated that structural modifications, enhanced active site plasticity, and optimized membrane positioning contribute to increased RhlB catalytic activity. Furthermore, we identified a critical role for ATP in RhlB function. Site-directed mutagenesis of the RhlB2 sequence revealed that a Histidine substitution at position 263 prevented M-RL production; reverting this residue to its original form restored enzymatic activity only when the Histidine was present, indicating ATP interaction via electrostatic forces.^65,94,95^ Structural modeling indicates that the predicted binding sites for dTDP-l-rhamnose and ATP are in close proximity; this suggests that ATP binding may promote a conformational shift toward an open, catalytically competent state, thereby facilitating donor substrate binding. This represents the first demonstration of energy nucleotide requirement for RhlB’s rhamnosylation reaction. However, direct biochemical characterization will be required to further confirm the enzymatic dependence on energy nucleotides. This study confirms that the integration of bioinformatics-driven mining and experimental validation provides an effective means to uncover naturally optimized variants of complex enzymes. This framework can be readily adapted for targeted enzyme engineering and enhancement of industrial biosurfactant production.

## Methods

### Bacterial Strains and Culturing Conditions

All bacterial strains used in this study are listed in Table S3. Cultures were initiated from single colonies and grown overnight in the appropriate medium supplemented with antibiotics. Strains were stored at −80 °C in 25% (v/v) glycerol. For both pUC19 and pBAD24 expression vectors, carbenicillin was used at a final concentration of 50 μg ml^–1^.

### Bioinformatic Mining Pipeline and Parameter Framework

The *P. aeruginosa* PAO1 strain was used as the bioinformatic reference.^56^ Reference *rhlB* DNA and its encoding protein sequences were obtained from the *Pseudomonas* Genome Database^96^ (Table S4), and active domains were annotated using I-TASSER (Iterative Threading ASSEmbly Refinement) and UniProt (Table S5). As of May 2020, the genomic data set comprised 5,850 *P. aeruginosa* and 2,246 *Pseudomonas* spp. genomes retrieved from the NCBI GenBank and the *Pseudomonas* Genome Database. The *rhlB* sequences were extracted, aligned, and clustered by sequence similarity to the PAO1 reference, with duplicates removed at both the nucleotide and protein levels.

To increase sequence variability, discontinuous MEGABLAST was applied instead of standard BLASTN or MEGABLAST, using a statistical significance threshold of 0.5 and a match/mismatch ratio of 1/–3.^97^ The mining framework consisted of four mutagenic cycles (250 sequences each cycle) utilizing a multilayer framework with defined hyperparameters for DNA and protein analysis. Sequences from each cycle were grouped by mutational characteristics, and representative candidates were iteratively used as inputs for subsequent cycles. Training models optimized the analysis parameters, incorporating mutational patterns and hotspot data to refine predictions. Experimentally validated data were then used to evaluate and calibrate the pipeline accuracy.

The pipeline was generated using Python (v3.8, 64-bit) within an Anaconda3 virtual environment in Visual Studio, with embedded scripts for automated heatmap visualization. The complete Python script is available at: https://github.com/PTrus2/MultiDomain-DNA-protein-mining-pipeline.git. At the nucleotide (nt) level, mining hyperparameters included mutation position (active domain, envelope, or encoding region), mutation type (silent or missense), premature stop codons, mismatch distribution, mutational hotspots/patterns, and base transversions or transitions.

Training models for the first four parameters were established prior to the initial cycle, whereas hotspot and mismatch pattern data were integrated from the first two cycles onward. Mutational hotspots were defined as silent or missense mutations shared by ≥4 sequences, while mutational patterns represented recurring nucleotide mismatches occurring at identical or conserved positions across sequence sets.

At the protein level, parameters describing function, tertiary structure, and active domain organization were obtained from the UniProt, EMBL-EBI, NCBI, and ScanProsite databases. Structural descriptors, including hydropathy, charge, polarity, and transmembrane positioning, were incorporated into the predictive models. Substrate and product binding parameters were developed using SwissDock^69,98^ with the PAO1 reference RhlB structure as a docking template (Tables S6 and S7). The AC score measured the total energy of the complex as the sum of force field and solvation energies, and the SwissParam score represented the binding free energies as the weighted sum of polar and nonpolar terms.^69,99,100^ Docking outputs were visualized in UCSF ChimeraX, and SMILES notations for HAAs, dTDP-l-rhamnose, ATP, GTP, and M-RL are listed in Table S8. RhlB binding cavity was detected using parKVFinder^101^ and cavity box coordinates were calculated using a custom Python script. The molecular docking box as seen in Figure S4 was generated from the custom script and visualized in ChimeraX. A grid box with dimensions of 18.7 × 20.9 × 15.3 Å was defined to encapsulate the entire binding cavity. Docking experiments were repeated 10 times testing various ligand orientations with conformations demonstrating the lowest AC score being selected for analysis. ATP binding motifs were mapped using TargetATP, ATPbind, ATPint, and NsitePred, and stability hotspots were identified using HotSpot Wizard 3.0.^102^ Protein models were generated via Swiss-Model,^103,104^ and intermolecular interactions (hydrophobic, hydrogen, and salt-bridge networks) were analyzed using ProteinTools.^105^ A penalty score matrix was constructed to quantify amino acid substitutions relative to substrate (dTDP-l-rhamnose, HAAs) and nucleotide (ATP/GTP) binding sites between the reference and variant sequences. Mutations located within five residues of a substrate binding site or three residues of an energy nucleotide binding motif were defined as spatially adjacent and functionally relevant.

### Sequence Alignment Algorithms

DNA and protein sequence alignments were performed using BLAST-based algorithms, including BLASTN, Discontinuous MEGABLAST, BLASTP, PSI-BLAST, DELTA-BLAST, and PHI-BLAST, primarily executed through BLAST+.^106^ Needleman–Wunsch and Clustal Omega algorithms were integrated into the mining workflow for pairwise and multiple sequence alignments, respectively. Phylogenetic alignment of RhlB sequences was carried out using MUSCLE within MEGA X.^107^ Evolutionary relationships among RhlB candidate sequences were inferred in MEGA X utilizing the Maximum Likelihood method with the Jones-Taylor-Thornton (JTT) matrix-based model and 1,000 bootstrap replicates.^107–109^ The analysis included 30 protein sequences, each comprising 426 amino acids.

### Genetic Constructs

The *rhlAB* operon was amplified from the *P. aeruginosa* genomic PAO1 genome using Phusion High-Fidelity DNA polymerase (NEB) and the primers listed in Table S12. The resulting 2.2 kb fragment was purified from a 0.7% agarose gel (Qiagen QIAquick Gel Extraction Kit). The *rhlAB* insert and pUC19 vector (NEB) were digested with *SphI* and *SacI*, purified, and ligated using T4 DNA ligase at a 1:3 vector-to-insert molar ratio. Ligated constructs were transformed into chemically competent *E. coli* DH5α cells (NEB). Positive transformants were screened by restriction digestion and confirmed by Sanger sequencing (Eurofins Genomics, Germany).

The modular *rhlAB* construct was designed with defined restriction sites between *rhlA* and *rhlB* to enable flexible gene replacement and configuration. The modular arrangement was designed as *Eco*RI*-rhlA-KpnI-rhlB-SphI*, with a 43 bp intergenic region based on the PAO1 *rhlAB* sequence. Seventeen base pairs separated the *rhlA* stop codon and *Kpn*I site, nine base pairs separated the restriction site from the *rhlB* Shine–Dalgarno sequence (AGGAGG), and five base pairs (AAACG) were positioned between the Shine–Dalgarno and *rhlB* start codon to maintain translational control.

The *rhlAB* operon from the *P. aeruginosa* PAO1 reference strain served as the template for the reference construct (Table S4), which was synthesized using the Thermo Fisher GeneArt service. The construct was subcloned into the pBAD24 expression vector (ATCC no. 87399). Variant *rhlB* sequences identified through the DNA–protein mining pipeline were synthesized (GeneArt) and cloned into the pBAD24-*rhlAB* reference backbone to generate the *rhlAB*1, *rhlAB*2, and *rhlAB*3 constructs (Figure S22). All constructs were verified by diagnostic restriction digestion and full-plasmid Sanger sequencing (Eurofins Genomics, Germany).

### Protein Induction for SDS-PAGE Electrophoresis and Polyacrylamide Gel Staining

RhlA and RhlB were induced and expressed in *E. coli* strains carrying pUC19-*rhlAB*, pBAD24-*rhlAB*, and pBAD24-*rhlAB* variant constructs. Protein concentrations were quantified using the Bradford assay (Pierce Protein Assay Kit, Thermo Fisher Scientific, UK) according to the manufacturer’s instructions. Samples (2.5 μg of total protein) were resolved on 12% stain-free precast SDS-polyacrylamide gels (Bio-Rad, UK) using a Mini-PROTEAN Tetra Cell system and a low-range molecular weight marker. Gels were stained with Imperial Protein Stain (Thermo Fisher Scientific, UK) for 1 h and visualized with a Bio-Rad ChemiDoc MP imager. Band intensities were analyzed using Bio-Rad Image Lab software. Three biological repeats were conducted to verify the size and expression of the *rhlAB* modular constructs.

### Gene Expression Analysis

pBAD24-*rhlAB* and *rhlB* variant constructs were induced using 0.7% arabinose and cultured for 18 h at 37 °C. Total RNA was extracted according to the manufacturer’s guidelines (Monarch Total RNA Miniprep Kit, T2010, NEB, UK). Total-RNA quality and concentration were measured using the Qubit 4 Fluorometer. *rhlB* expression levels were quantified using the Luna Universal One-Step RT-qPCR Kit (E3005S, NEB, UK) and the Applied Biosystems QuantStudio 6 Pro real-time PCR system. *rhlB* calibration curve was established using the *rhlAB* reference construct utilizing concentrations from 0.01 to 1,000 ng; a concentration of 10 ng was used for all *rhl*B variant analyses. For the 16S housekeeping gene, a 1:50 dilution was used for data normalization between all samples. Gene expression analysis was performed using Diomni Design and Analysis qPCR software. Three biological replicates, each measured in triplicate, were used to evaluate *rhlB* variant gene expression.

### Victoria Blue Colorimetric Assay

The Victoria Blue (VB) assay, adapted from Kubicki et al.,^61^ was used to quantify M-RL production. VB stock solution (Acros Organics, US; 1 mg mL^–1^ in isopropanol) was diluted to 0.1 mg mL^–1^, and 96-well microplates were coated and fixed as previously described. Prepared plates were stored at 4 °C until use. Commercial rhamnolipids (Agae Technologies, US) were used to generate calibration standards (0–1 mg mL^–1^). For host-derived background correction, cell-free supernatant from *E. coli* DH5α carrying an empty pBAD24 vector, grown in ESM with carbenicillin (50 μg mL^–1^), served as the reference. Rhamnolipid standards or filtered cell-free culture supernatants (200 μL per well) were incubated in triplicate at 25 °C and 250 rpm for 1 h. Absorbance was measured at 625 nm by using a Biotek Synergy HT multimode reader. M-RL production from *rhlAB* clones was determined from three biological replicates, each measured in triplicate under the respective IPTG or arabinose induction conditions.

### Oil Diffusion Assay

The oil diffusion assay was performed using paraffin oil dyed with 0.5% (w/v) Sudan Black B (Thermo Fisher Scientific, UK) as a visual indicator. Forty milliliters of distilled water were added to a 150 mm Petri dish, and a 10 μL droplet of dyed paraffin oil was placed on the surface. A 1 μL aliquot of bacterial supernatant was then applied to the center of the oil droplet. Biosurfactant activity was indicated by the displacement of the oil and the formation of a clear diffusion zone.

### Contact Angle Measurements

Contact angle analysis was conducted to validate the accuracy of the Victoria Blue assay. Commercial RL were diluted in ESM to concentrations of 0–1 mg mL^–1^ to generate a standard curve. For each sample, 8 μL droplets were placed on a polydimethylsiloxane (PDMS) surface affixed to the sample stage to provide a uniform hydrophobic substrate. Droplet images were captured using a 5-megapixel camera. Each condition was analyzed in triplicate, and all measurements were performed across three biological replicates to determine standard deviations. Contact angles (θ) were calculated from droplet dimensions based on the following equation:

1 $$\theta =2\times arc\tan\left(\frac{H}{R}\right)$$

where, *H* is the droplet height and *R* is the base radius.

### Statistical Analysis

All statistical analyses were performed using GraphPad Prism (version 10.5.0) and Microsoft Excel. The choice of statistical tests depended on data distribution and is listed as follows: one-way ANOVA in conjunction with Tukey, Šidák, Dunnett, and Bonferroni multiple comparison tests, two-tailed Welch’s *t* test, descriptive statistics, and simple linear regression. All experiments consisted of three biological replicates, each measured in technical triplicate. GraphPad Prism was also used to generate graphs for this study. Limit of detection (*LOD*) was calculated using the following equation:

2 $$LOD=\frac{3.3\sigma }{S}$$

where σ is the standard deviation of the response, *S* is the slope of the calibration curve, and 3.3 is a statistical constant used to estimate the detection limit.

## Supplementary Material



## Notes

Additional experimental results (Figures S1–S21; Tables S1 and S2); supplementary epidemiological and bioinformatic discussion; supplementary materials and methods (Figure S22; Tables S3–S12) supporting the results and discussion (PDF)

## Abbreviations

RL: rhamnolipids
M-RL: monorhamnolipids
Di-RL: dirhamnolipids
nt: nucleotide/s
HAAs: 3-(3-hydroxyalkanoyloxy) alkanoic acids
ATP: adenosine triphosphate
GTP: guanosine triphosphate
VB: Victoria Blue
LB: Luria–Bertani
ESM: *E. coli* Screening Medium
LOD: limit of detection
MAPE: mean average percentage error
CF: cystic fibrosis
bp: base-pair
kb: kilobase pair
SDS-PAGE: sodium dodecyl sulfate-polyacrylamide gel electrophoresis
Å: angstrom
JTT: Jones-Taylor-Thornton
MMR: methyl-directed mismatch repair
