# Supplementary material for: Multidomain DNA–Protein Mining Reveals Polymorphic Variations in RhlB Enhancing Monorhamnolipid Biosynthesis
Source: ACS Synth Biol. 2026 Apr 17;15(5):1831–49. doi: 10.1021/acssynbio.5c00828 (PMC13185158; doi:10.1021/acssynbio.5c00828)
Supplement: Supplementary file 1 [file sb5c00828_si_001.pdf]

Supporting Information

Multidomain DNA–protein mining reveals polymorphic variations in RhlB enhancing mono-rhamnolipid biosynthesis

Pavlos Trus[1,2], Chien-Yi Chang[1,2]*

[1]School of Dental Sciences, Faculty of Medical Sciences, Newcastle University, Newcastle Upon Tyne, NE2 4BW, UK

[2]Biosciences Institute, Faculty of Medical Sciences, Newcastle University, Newcastle Upon Tyne, NE2 4HH, UK

*Corresponding author: Chien-Yi Chang

Email: chienyi.chang@newcastle.ac.uk

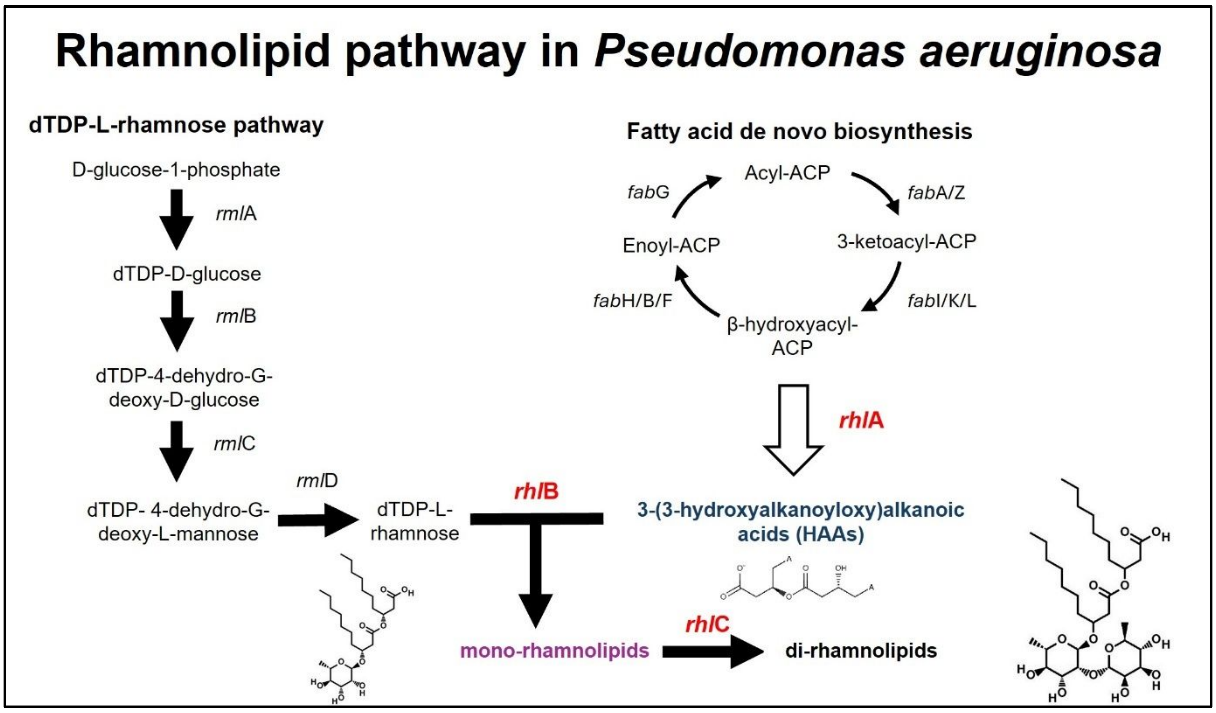

Figure S1. Rhamnolipid biosynthesis in *P. aeruginosa*. Two main pathways feed into rhamnolipid biosynthesis, first the dTDP-L-rhamnose pathway provides the sugar moiety, while the fatty acid de novo biosynthesis pathway supplies the fatty acid building blocks in the form of β-hydroxyacyl-ACP. Rhamnolipid biosynthesis is governed by three key genes located in two distinct operons *rhlAB* and *rhlC*. Enzymatic function: RhlA converts β-hydroxyacyl-ACP into 3-(3-hydroxyalkanoyloxy) alkanoic acids (HAAs), RhlB catalyses the first rhamnosylation reaction forming mono-rhamnolipids, in which dTDP-L-rhamnose is covalently attached to HAAs through an α-glycosidic linkage. RhlC catalyses the second rhamnosylation reaction which adds an additional dTDP-L-rhamnose moiety forming di-rhamnolipids. Chemical structures for HAAs, mono- and di-rhamnolipids can be shown within the figure.

Nucleotide analysis parameters: Mutation position and type

A

Step 1: *rhl*B

Polymorphic variations were analysed based on active domain localisation

Nucleotide sequence segments	nt position
1. Active domain **A**	p10 – 405
2. Active domain **B**	p691 – 1141
3. Envelope **A**	p4 – 10 & p405 – 414
4. Envelope **B**	p607 – 691 & p1141 – 1221
5. Non- Active domain/ Envelope	p1 – 4, p414 – 607 & p1221 – 1281

→

Sequences were categorised based on polymorphic position within the nt sequence

Group 1: Envelope **A** + Non- Active domain/ Envelope

Group 2: Envelope **B** + Non- Active domain/ Envelope

Group 3: Active Domain **A**

Group 4: Active Domain **B**

Group 5: Envelope **A**

Group 6: Envelope **B**

Group 7: Envelope **A** + Active domain **A**

Group 8: Envelope **B** + Active domain **B**

Group 9: Envelope + Active domain **A** + Non- Active domain/ Envelope

Group 10: Envelope + Active domain **B** + Non- Active domain/ Envelope

Type of mutations

B

Step 2: Mutational analysis

Polymorphic variations were categorised based on encoding codons

1. Sequences were separate into three-letter nucleotides	→	Defined from first ATG (start-codon) and last TGA (stop codon)
2. Sequences separated into start- **encoding region** -stop	→	Encoding region defined as three-letter nt after start and before stop codons
3. Sequences removed displaying premature stop-codons	→	Defined as TAA, TGA and TAG within encoding region
4. Nucleotide mutations	→	Defined as 1 nt, 2 nt or 3 nt changes within encoding codons
5. Mutations categorised as silent mutations	→	Defined as 1 nt or 2 nt changes that don't change amino acid grouping
6. Mutations categorised as substitutions	→	Defined as 1 nt, 2 nt or 3 nt changes that leads to an amino acid substitution
7. Sequences grouped based on silent mutations	→	Defined as **group A:** shared mutation and **group B:** not shared
8. Sequences grouped based on substitutions	→	Defined as **group C:** shared mutation and **group D:** not shared

Figure S2. Nucleotide sequence analysis parameters. Analysis parameters for *rhlB* written in pseudocode. (A) Step 1: mutation distribution with functionally important segments (active domain A & B, domain envelopes, and non-active domain and envelope). (B) Step 2: type of mutation analysis (silent or missense) withing gene encoding region. Data from step 1 and step 2 were used to build the mutational pattern and mutation hotspot analysis parameters.

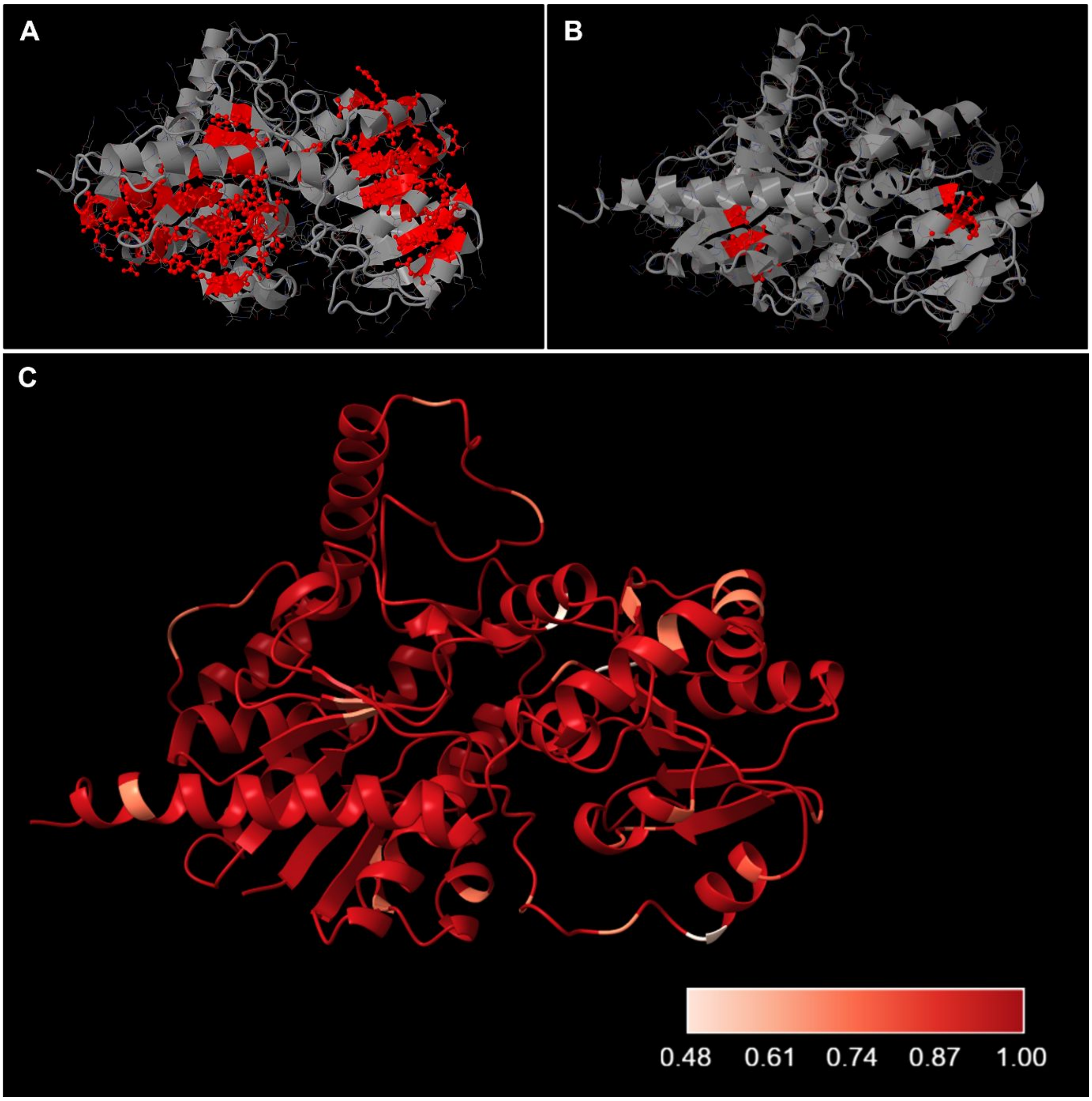

Figure S3. RhlB stability hotspots and conservation analysis. (A) Total stability hotspots identified: 35, based on majority and frequency ratio consensus approach. (B) Stability hotspots with the highest probability of contributing to protein folding and structure flexibility (10 hotspots). (C) Conservation analysis of phylogenetic candidate sequences in relation to total stability hotspots. Heatmap scale illustrates evolutionary rate from low to high amino acid conservation. Input sequences were selected based on mutation proximity (1 to 3 amino acids) to stability hotspots. (A) & (B) models were created using Hotspot Wizard and (C) was illustrated using ChimeraX. Conservation analysis script is included in the protein pipeline.

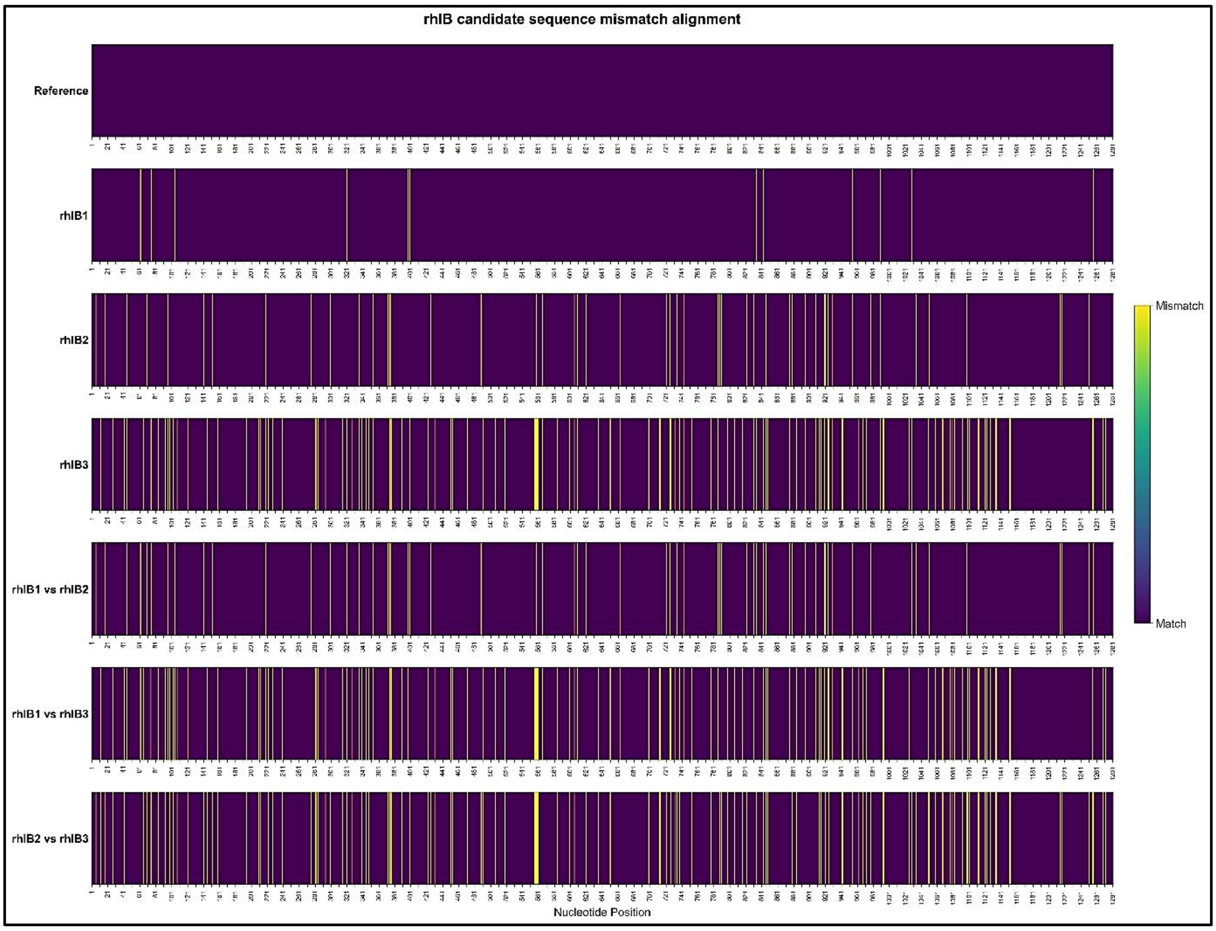

Figure S4. Nucleotide mismatch alignment heatmap between the reference *rhlB* and candidate sequences. Reference *rhlB* sequence does not display any mismatches (control alignment). Each of the candidate sequences (*rhlB*1, *rhlB*2, *rhlB*3) were compared to the reference *rhlB* and to each other (*rhlB*1 versus *rhlB*2, *rhlB*1 versus *rhlB*3*,* and *rhlB*2 versus *rhlB3*). Areas that demonstrate an increase in colour intensity correspond to nucleotide mutation hotspots. Mismatches correspond to nucleotide sequence positions.

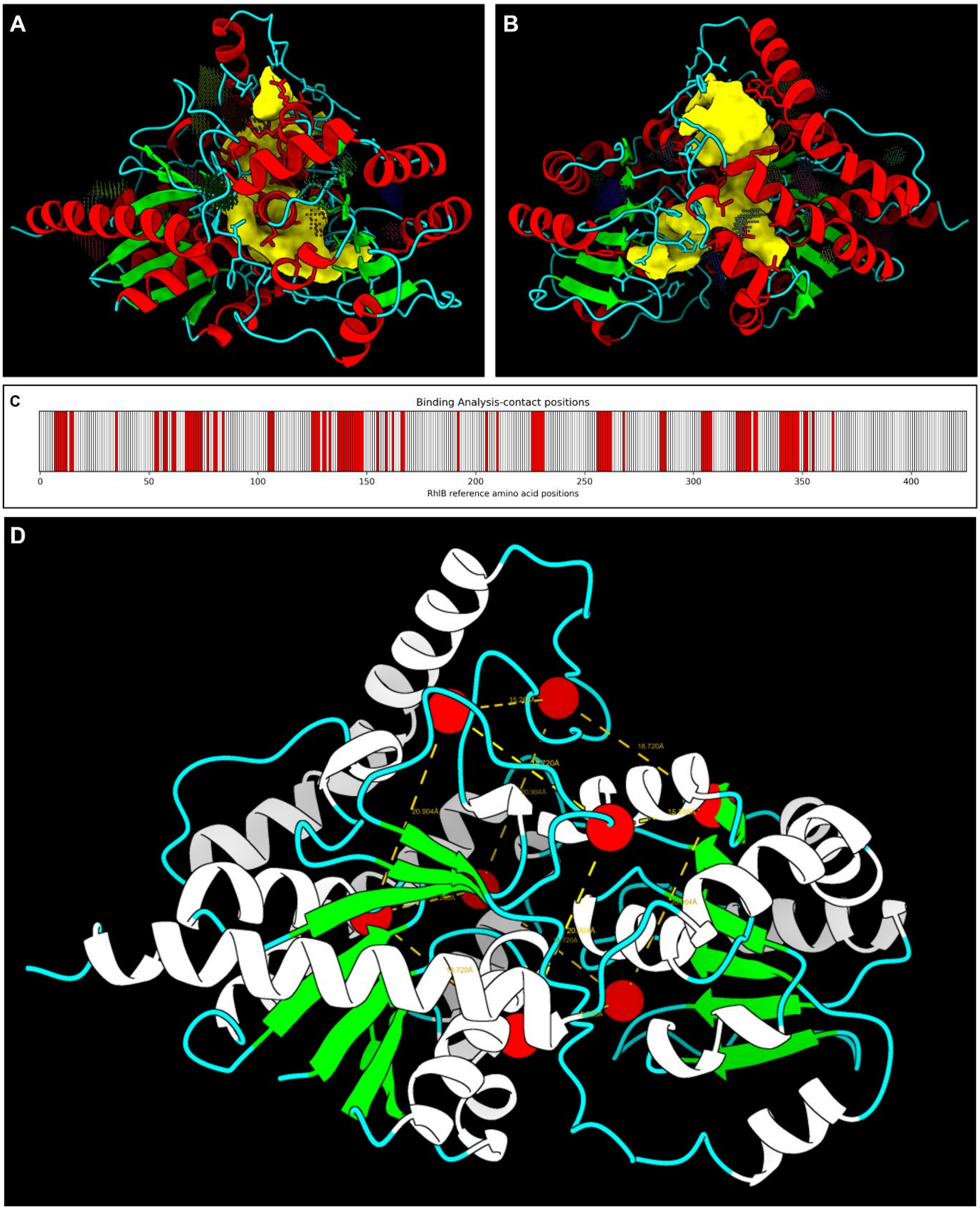

Figure S5. RhlB biomolecular cavity analysis. Binding cavities detected using parKVFinder in ChimeraX utilising the reference RhlB model as input. (A) Binding cavity displaying highest binding affinity coloured yellow. (B) 180-degree horizontal rotation. (C) Contact residue heatmap in relation to the reference RhlB sequence. (D) Molecular docking box calculated and visualised from finding cavity analysis.

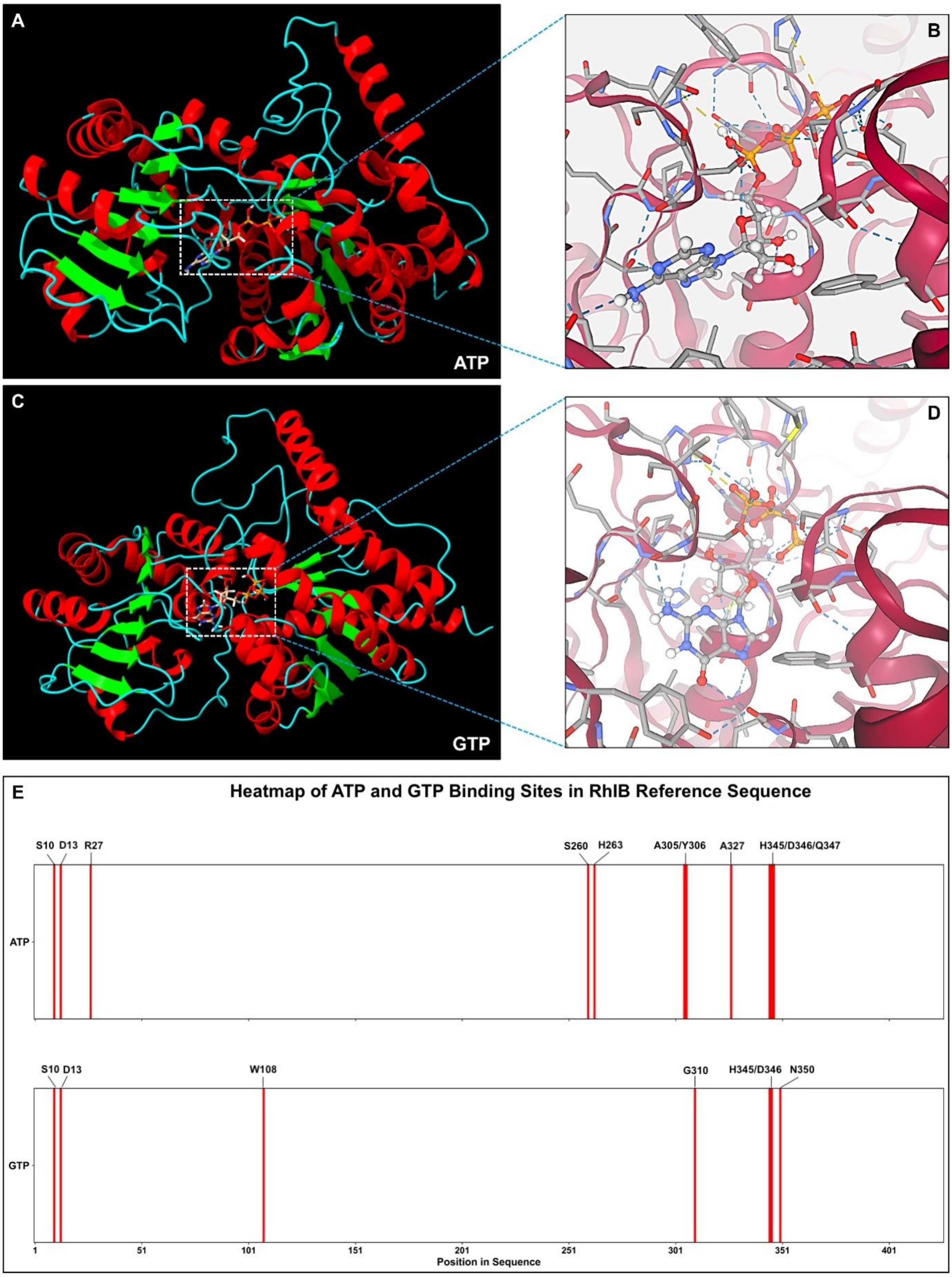

Figure S6. Position of ATP and GTP within RhlB. (A) ATP and (C) GTP positions relative to the reference RhlB tertiary structure, while (B) ATP and (D) GTP protein structures demonstrate the intermolecular forces between the protein structure and each ligand. (E) Heatmap of ATP and GTP binding site positions mapped onto the RhlB_ref sequence, with labels indicating the corresponding amino acid residues and positions.

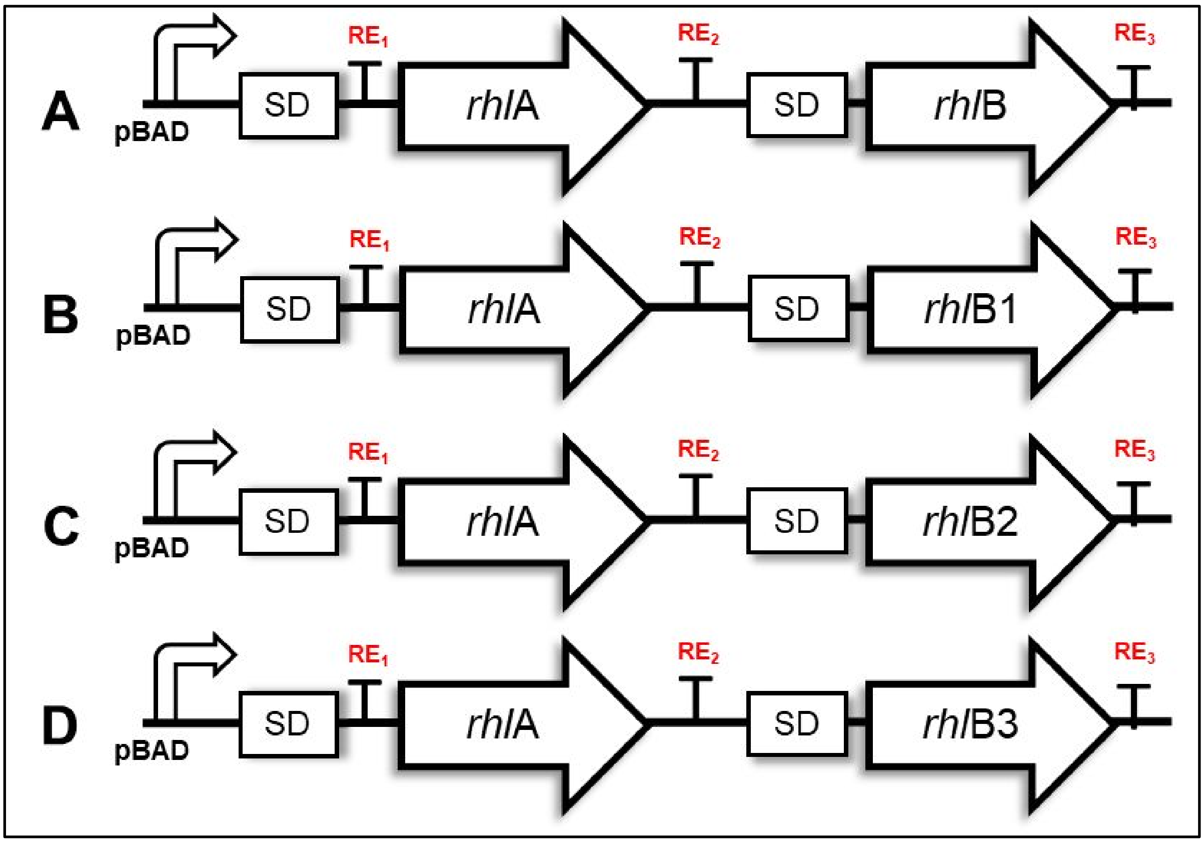

Figure S7. Modular genetic circuit of the M-RL pathway using an arabinose induction platform. (A) Modular construct with the reference *rhlA* and *rhlB* genes derived from the PAO1 strain. (B) RhlB1 modular construct containing the first selected *rhlB* sequence with protein identity of 99.53% with the reference model. (C) RhlB2 modular construct with the second *rhlB* sequence with protein identity of 97.65% with the reference model. (D) RhlB3 modular construct with the third selected *rhlB* sequence with protein identity of 94.13% identity with the reference model. Modular construct genetic elements: pBAD: arabinose promoter, SD: Shine-Dalgarno sequence, restriction enzyme (RE), RE_1: *EcoRI*, RE_2: *KpnI*, RE_3: *SphI*, arrows represent genes (*rhlA* and *rhlB*) and orientation. Modular construct size: 2,227 bp.

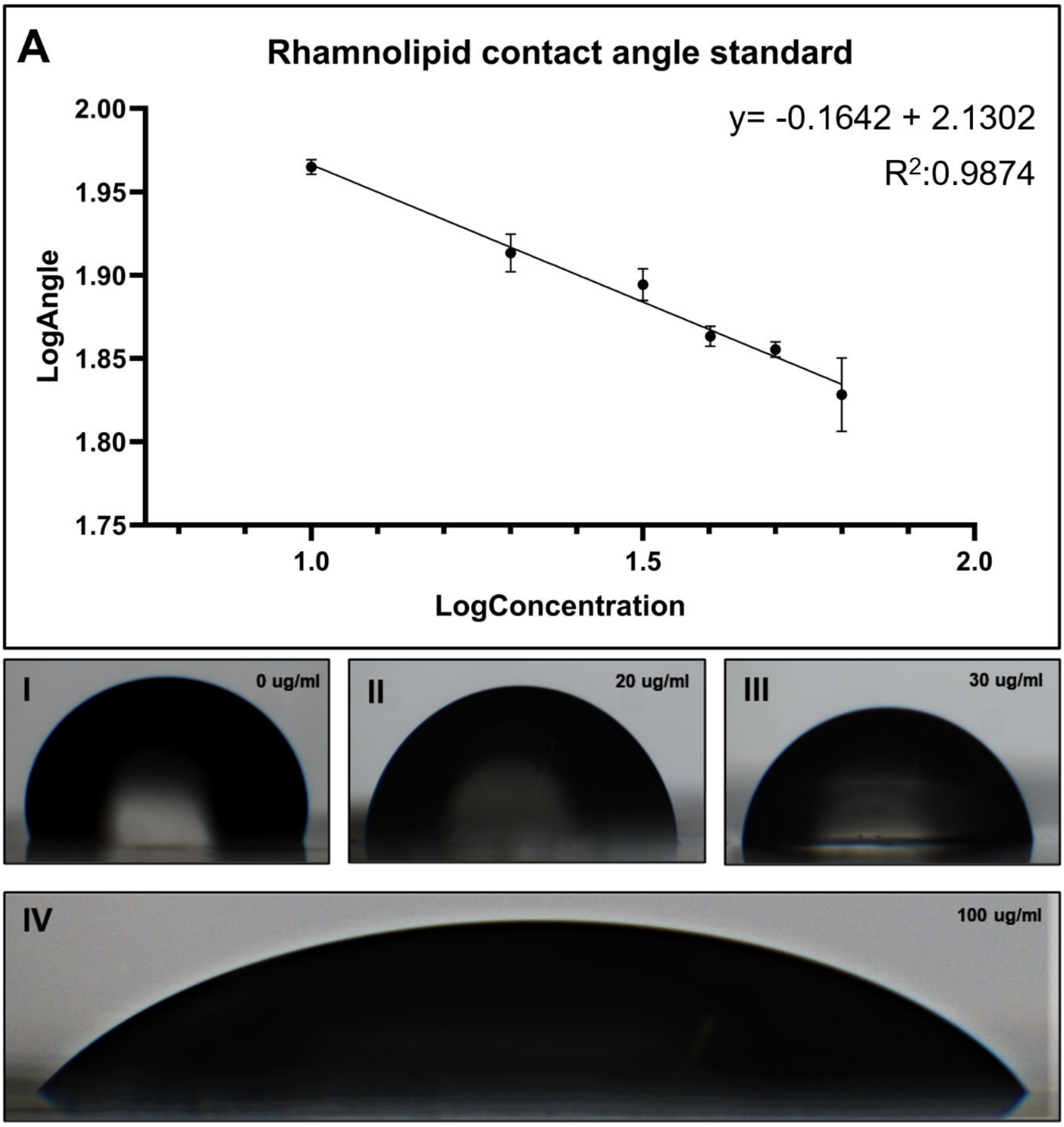

Figure S8. Rhamnolipid contact angle standard. (A) commercial RL contact angle standard in ESM media in log format. The concentrations of RL (logarithmic scale in bracket) ranged from 10 (1), 20 (1.3), 30 (1.5), 40 (1.6) 50 (1.7) and 60 (1.8) µg ml^{-1}. Images I, II, III, IV show a representation of the droplets on the PDMS surface. I: droplet at 0 µg ml^{-1} with a contact angle of 103.27º, II: droplet at 20 µg ml^{-1} with a contact angle of 81.91º, III: droplet at 30 µg ml^{-1} with a contact angle of 78.4º and IV: droplet at 100 µg ml^{-1} with a contact angle 54.67º. All droplets were 8 µl in size and captured in triplicate using a 5-megapixel camera.

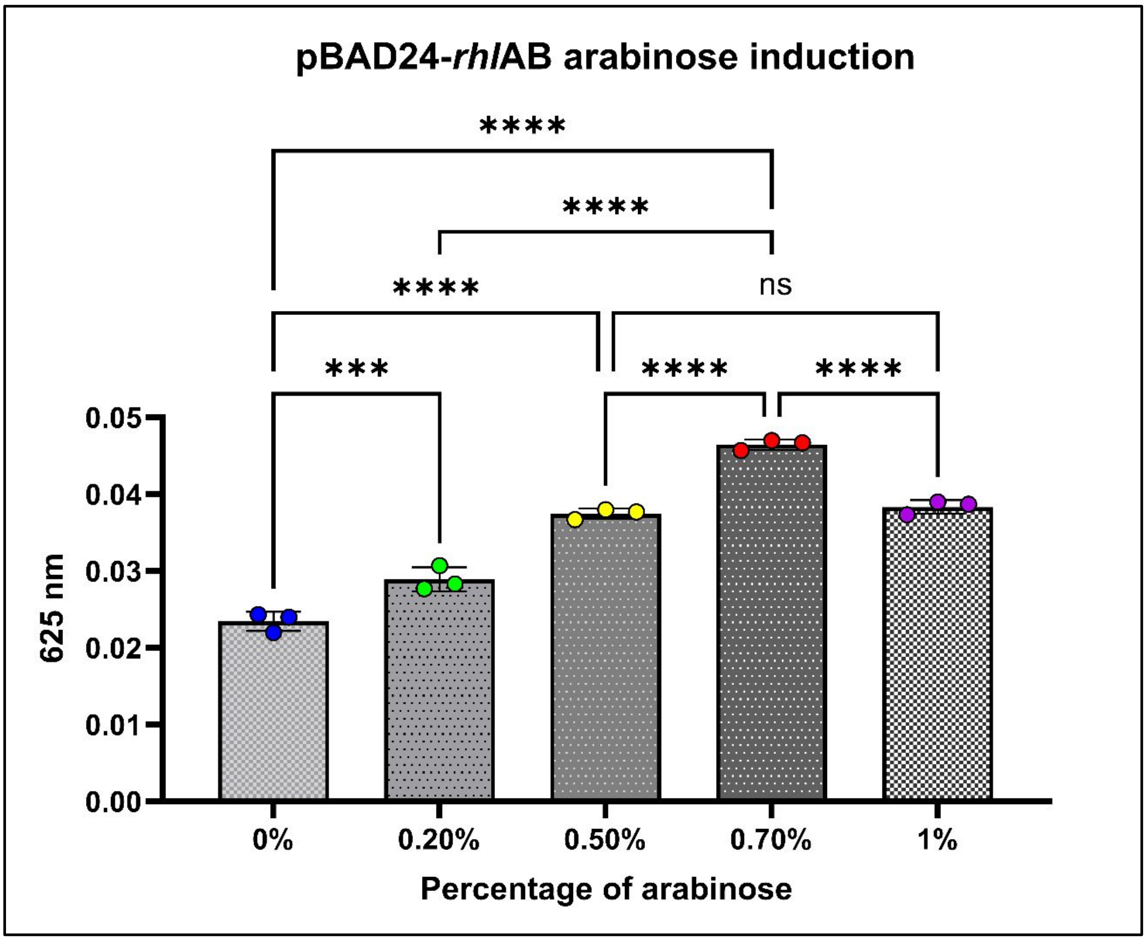

Figure S9. Determining arabinose concentration for *araBAD* promoter induction. The reference pBAD24-rhlAB was grown in ESM using increasing concentrations of arabinose 0%, 0.2%, 0.5%, 0.7%, 1% (w/v) and evaluated using the Victoria Blue assay. Samples were analysed using one-way ANOVA coupled with Tukey's multiple comparison test. Victoria Blue absorbance: 625 nm wavelength. ns: 0.1234, (***): 0.0002, (****): <0.0001.

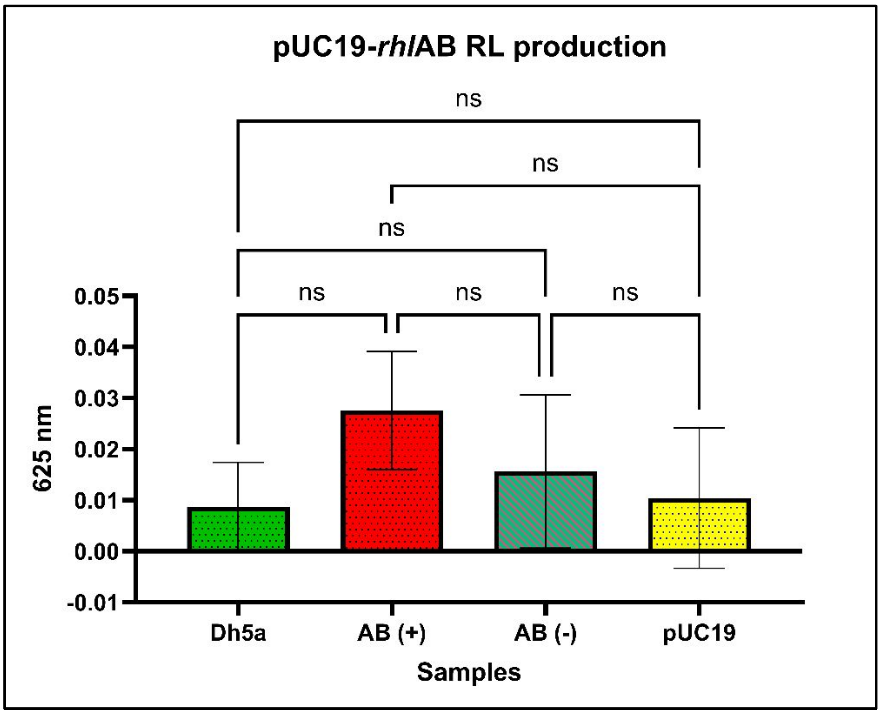

Figure S10. pUC19-*rhlAB* reference construct rhamnolipid biosynthesis using LB media supplemented with 4% glucose and 1% glycerol. RL production was quantified using the Victoria Blue assay, absorbance was measured at 625 nm wavelength. Experimental controls for this assay were the bacterial host (DH5a) and the empty pUC19 plasmid. The induced pUC19-*rhlAB* is represented as AB (+), while the non-induced clone as AB (-). Five biological repeats were performed for this experiment using fresh media. There was no significant difference between samples. Samples were statistically analysed using one-way ANOVA coupled with Tukey's multiple comparison test. ns> 0.1234.

Table S1. Rhamnolipid oil diffusion standard

**RL concentration**	1 pg ml^{-1}	100 pg ml^{-1}	1000 pg ml^{-1}	10,000 pg ml^{-1}	100,000 pg ml^{-1}	1 µg ml^{-1}
**Time (s)**	100s	68s	44s	23s	9s	1s
**Diameter**	2 mm	2 mm	3 mm	5 mm	10 mm	90 mm

Table S2. Oil diffusion screening of *E. coli* induction media

**pUC19-*rhl*AB**	Media 1	Media 2	Media 3	Media 4	Media 5	Media 6	Sup. LB
Diffusion	+	+	+	+	+	+	+
**DH5α**	Media 1	Media 2	Media 3	Media 4	Media 5	Media 6	Sup. LB
Diffusion	-	-	-	+	-	-	+
**pUC19**	Media 1	Media 2	Media 3	Media 4	Media 5	Media 6	Sup. LB
Diffusion	-	-	-	+	-	-	+
**Stock solutions**	M9 media	LB media	Tryptone	Peptone	Yeast extract	Casamino acids	Sup. LB
Diffusion	+	+	+	+	-	+	+

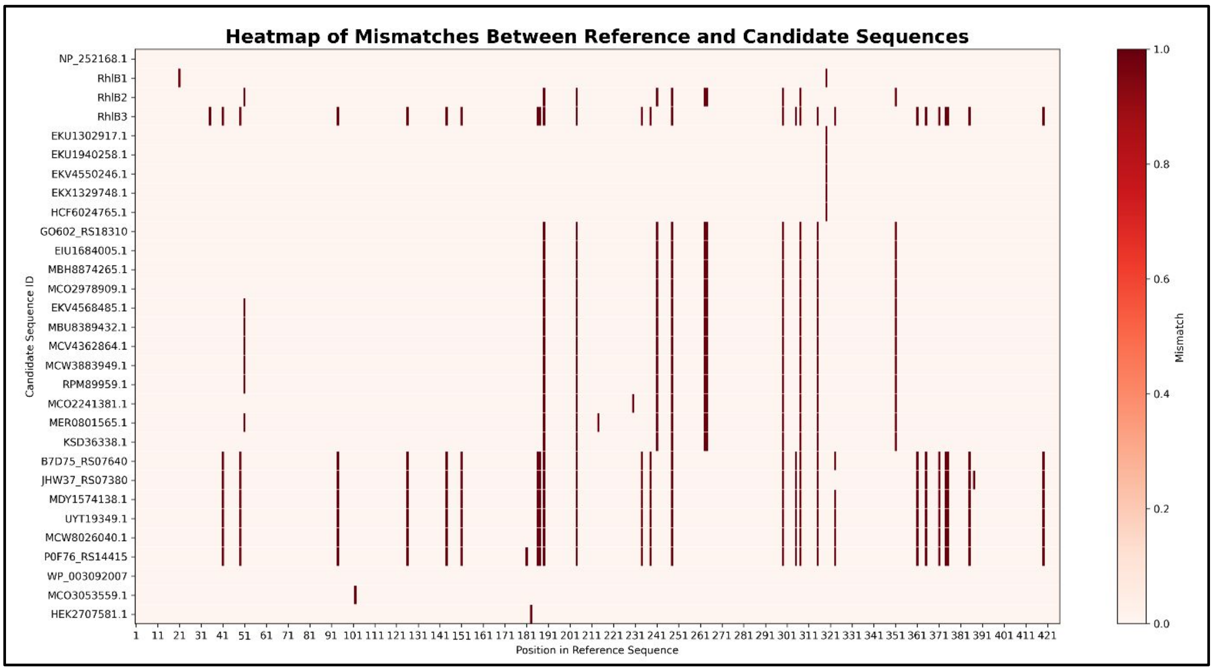

Figure S11. Mismatch heatmap between phylogenetic tree candidate RhlB sequences and reference RhlB sequence. Amino acid substitutions are illustrated in red compared to their position in the reference sequence. Increase in heatmap intensity indicates a substitution cluster (e.g. p186, p263 and p374).

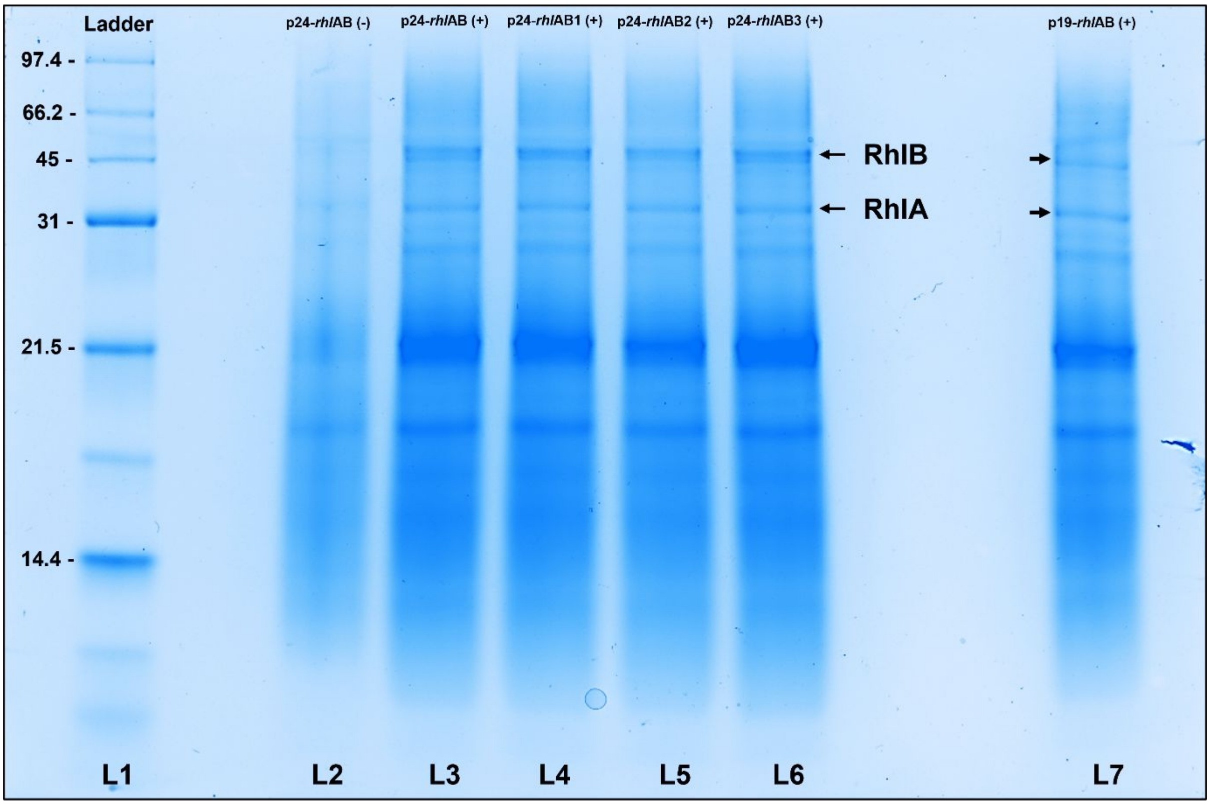

Figure S12. Protein SDS-PAGE electrophoresis. Expression and analysis of RhlAB proteins using Coomassie brilliant blue staining. L1: Bio-Rad low-range SDS-PAGE molecular weight protein ladder in kDa, L2: uninduced reference pBAD24-*rhlAB* sample, L3: induced reference pBAD24-*rhlAB* sample, L4: induced pBAD24-*rhlAB*1 sample, L5: induced pBAD24-*rhlAB*2 sample, L6: induced pBAD24-*rhlAB*3 sample, L7: induced reference pUC19-*rhlAB* sample. SDS-page was analysed using the Bio-Rad Image Lab software: version 6.1. Molecular weight: RhlA: 32.836 kDa, RhlB: 47.126 kDa. Induced samples are represented with a "+" and non-induced with a "-".

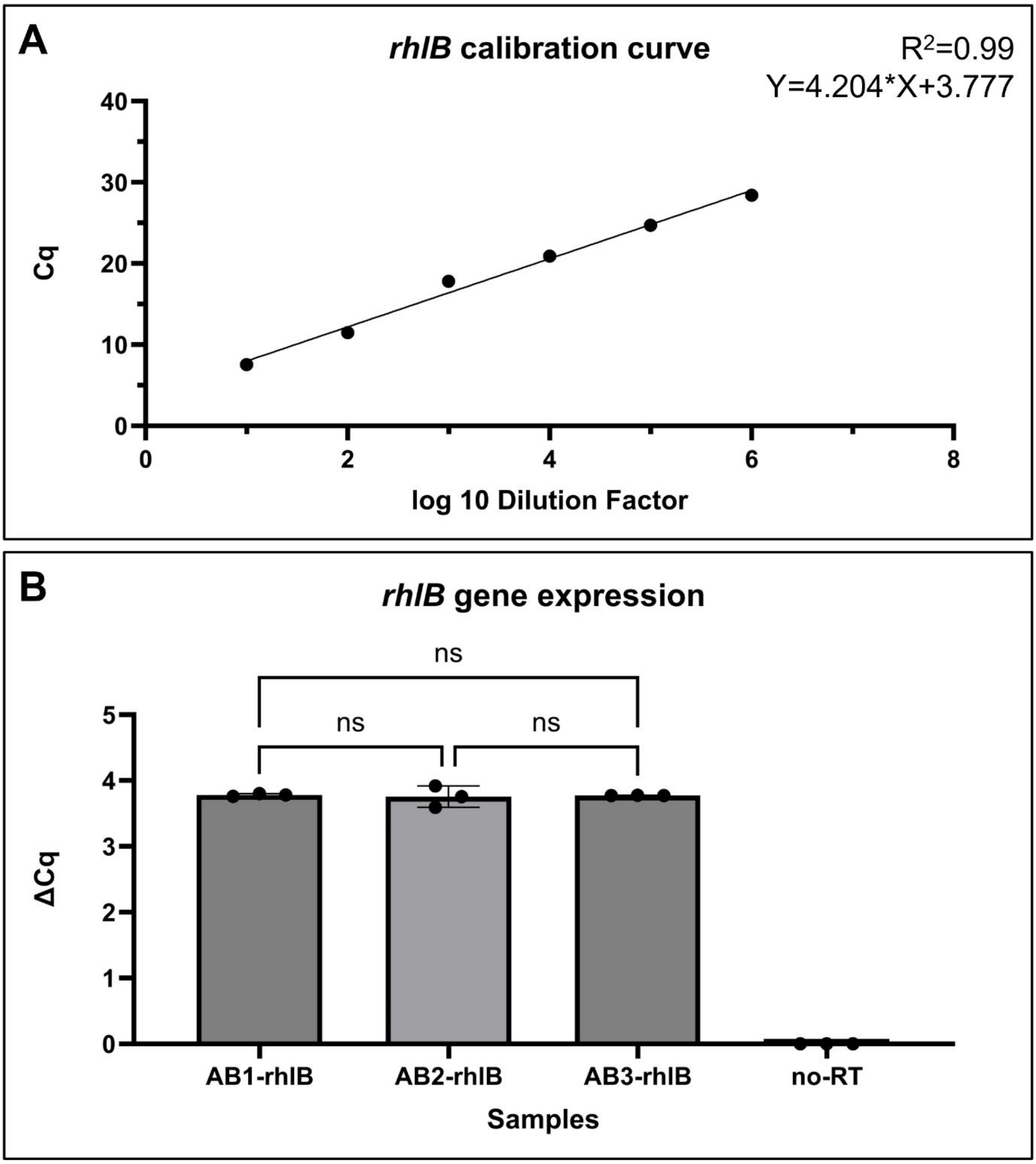

Figure S13. Gene expression analysis of *rhlB* variant constructs. (A) *rhlB* calibration curve utilising the reference pBAD24-AB construct Cq values and a total-RNA concentration (ng) range (0.01, 0.1, 1, 10, 100 and 1000) in log_{10} dilution factor format. (B) ΔCq *rhlB* gene expression analysis of each *rhlB* variant construct (pBAD24- AB1, AB2 and AB3) normalised against the housekeeping gene (16S). Data points were statistically analysed using one-way ANOVA coupled with Sidak's multiple-comparison test. Negative control (no-RT): reaction omitting the reverse transcriptase enzyme. ns> 0.1234.

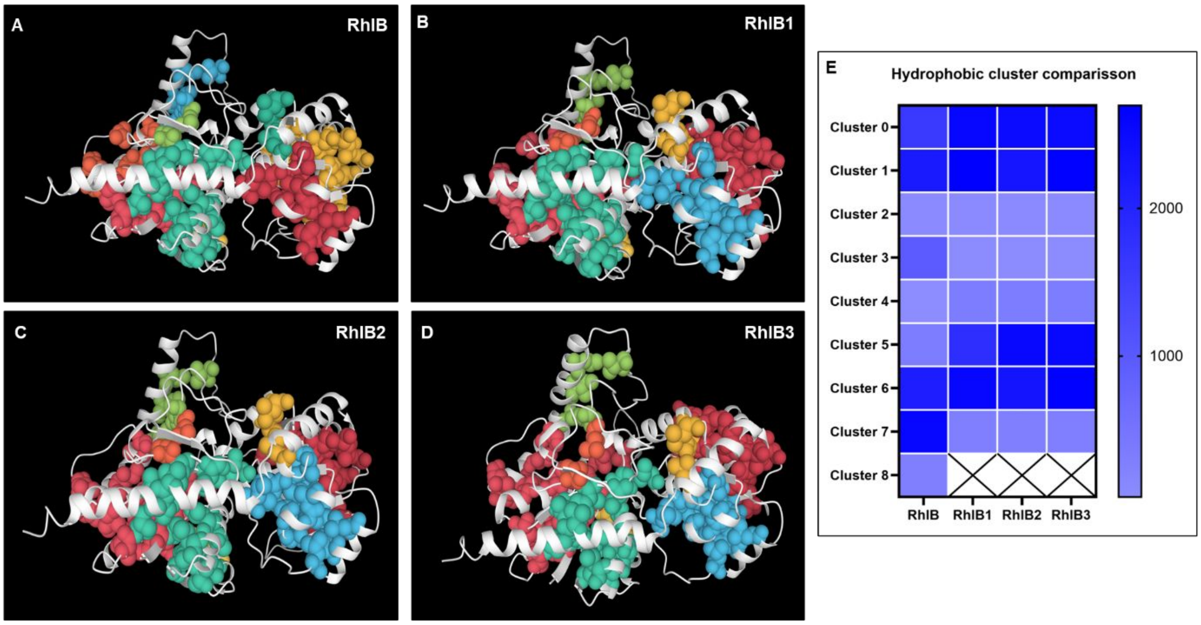

Figure S14. Hydrophobic network comparison. Graphical representation of hydrophobic network within the RhlB protein structures: (A) reference RhlB protein model, (B) RhlB1 model, (C) RhlB2 model, (D) RhlB3 model. Spheres represent atoms and same colour spheres make up the hydrophobic clusters. (E) Heat-map comparing the hydrophobic clusters between each RhlB protein model, comparison based hydrophobic cluster total areas.

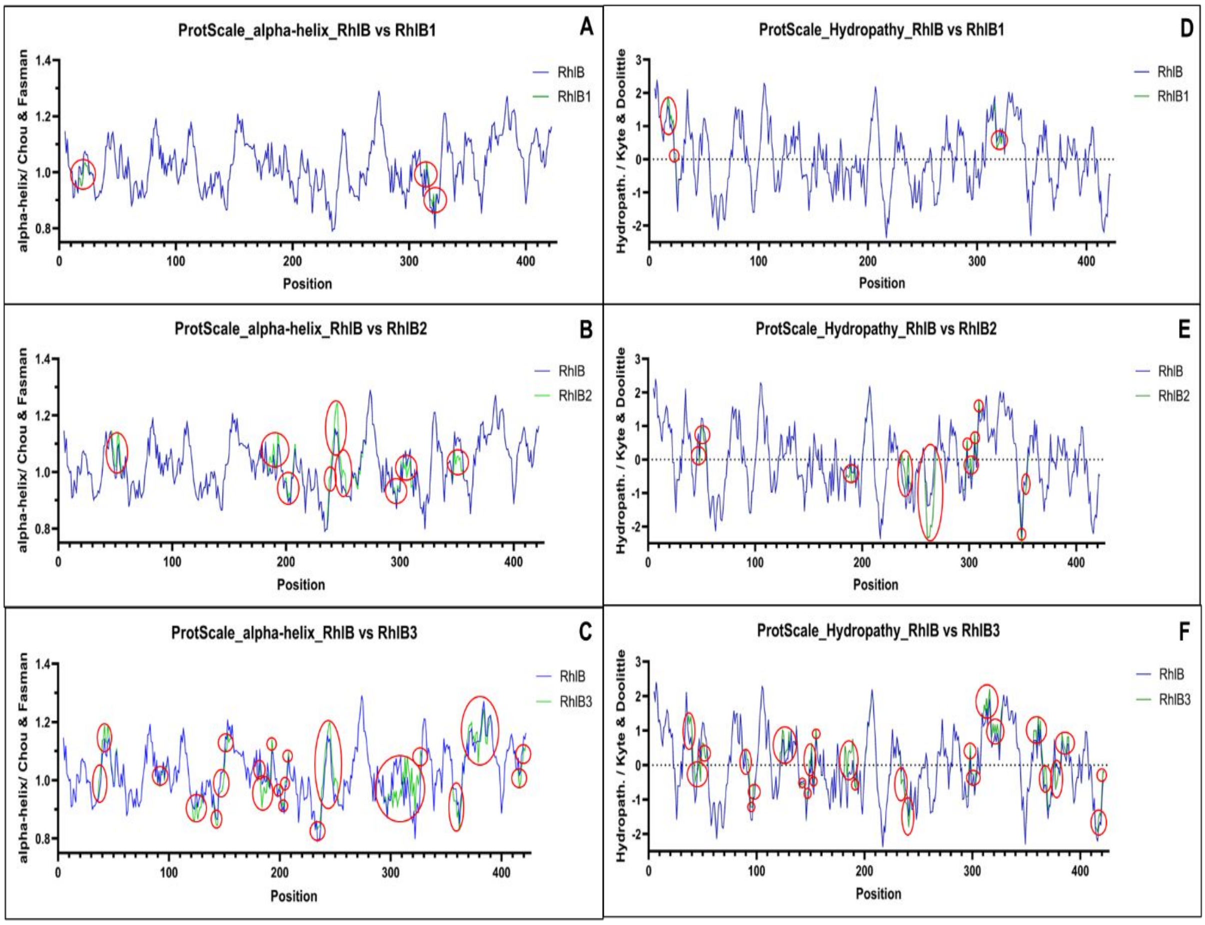

Figure S15. Comparing the α-helicase conformational parameters and hydropathy profile between reference RhlB (RhlB_ref) model and the selected RhlB (1, 2 & 3) models. (A–C) Comparison of α-helicase conformational parameters using the Chou & Fasman scale. (A) Comparison of RhlB_ref with RhlB1. (B) Comparison of RhlB_ref with RhlB2. (C) Comparison of RhlB_ref with RhlB3. (D–F) Comparison of hydropathy profile using the Kyte & Doolittle scale. (D) Comparison of RhlB_ref with RhlB1. (E) Comparison of RhlB_ref with RhlB2. (F) Comparison of RhlB_ref with RhlB3. In all graphs RhlB_ref is represented as a blue line and the experimental RhlB models (1, 2, and 3) with a green line. The red circles represent areas demonstrating differences between the models.

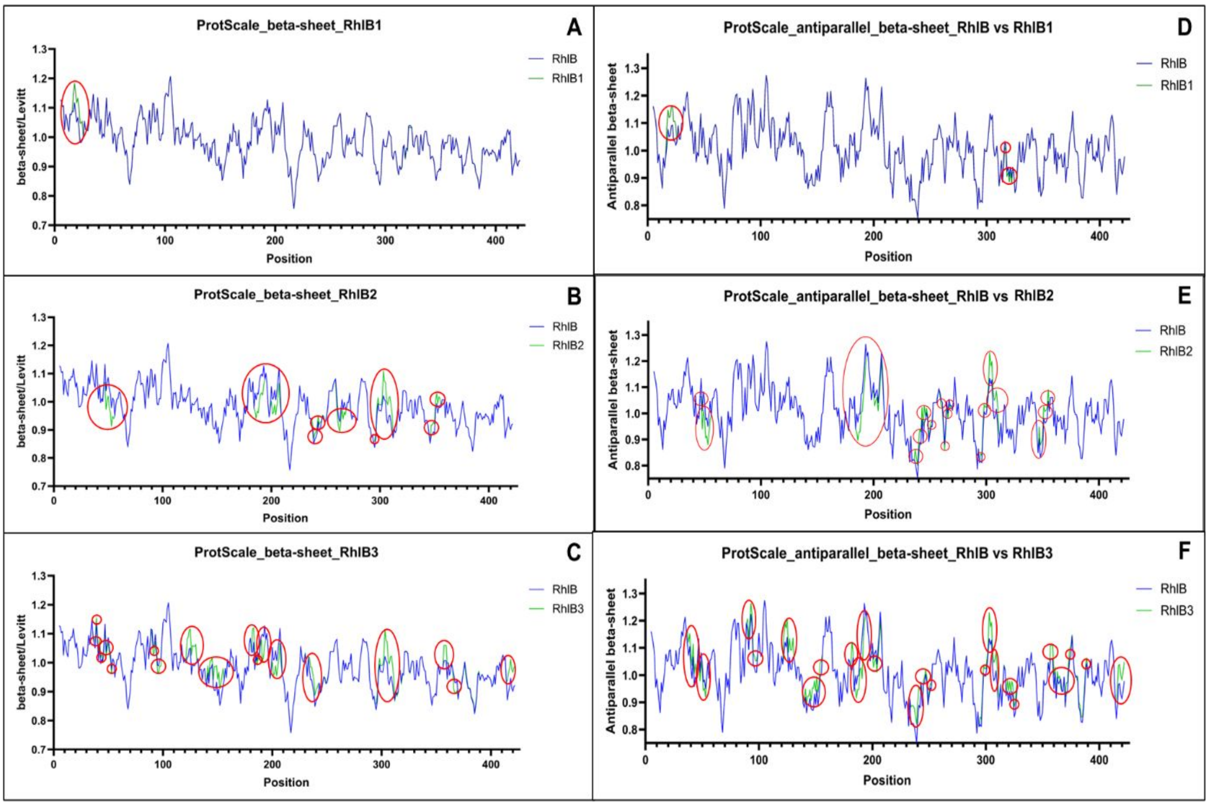

Figure S16. Comparison of β-sheet and antiparallel β-sheet between the reference RhlB (RhlB_ref) model and the selected RhlB (1, 2, and 3) models. (A–C) Comparison of β-sheet conformational parameters using Levitt scale. (A) Comparison between RhlB_ref and RhlB1. (B) Comparison between RhlB_ref and RhlB2. (C) Comparison between RhlB_ref and RhlB3. **(**D–F) Comparison of antiparallel β-sheet conformational preferences using the Lifson and Sander scale. (D) Comparison between RhlB_ref and RhlB1. (E) Comparison between RhlB_ref and RhlB2. (F) Comparison between RhlB_ref and RhlB3. RhlB_ref is illustrated as a blue line while the experimental RhlB models (1, 2, and 3) are displayed in green. The red circles represent the differences between the experimental models and the reference model.

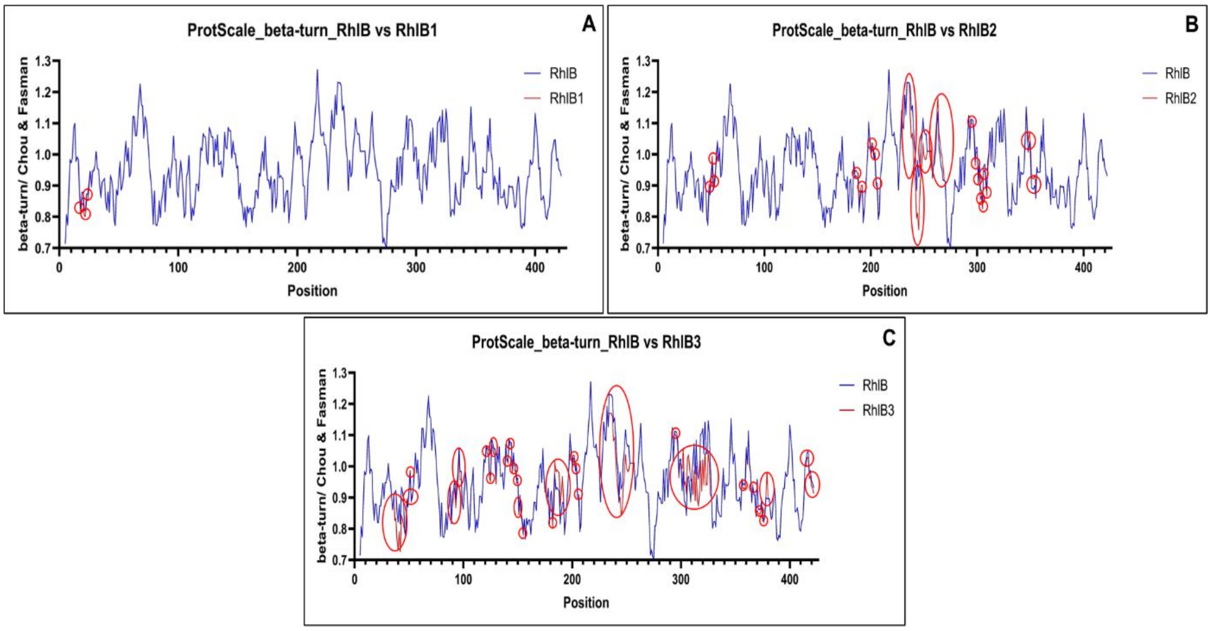

Figure S17. Comparison of β-turn profile between the reference RhlB (RhlB_ref) model and the selected RhlB (1, 2, and 3) models. (A) Comparison between RhlB_ref and RhlB1. (B) Comparison between RhlB_ref and RhlB2. (C) Comparison between RhlB_ref and RhlB3. The β-turn conformational parameters between each experimental RhlB model and the RhlB_ref model was analysed using the Chou and Fasman scale. The RhlB_ref profile is represented using a blue line and the experimental RhlB models (1, 2, and 3) using red. The red circles represent differences between the models.

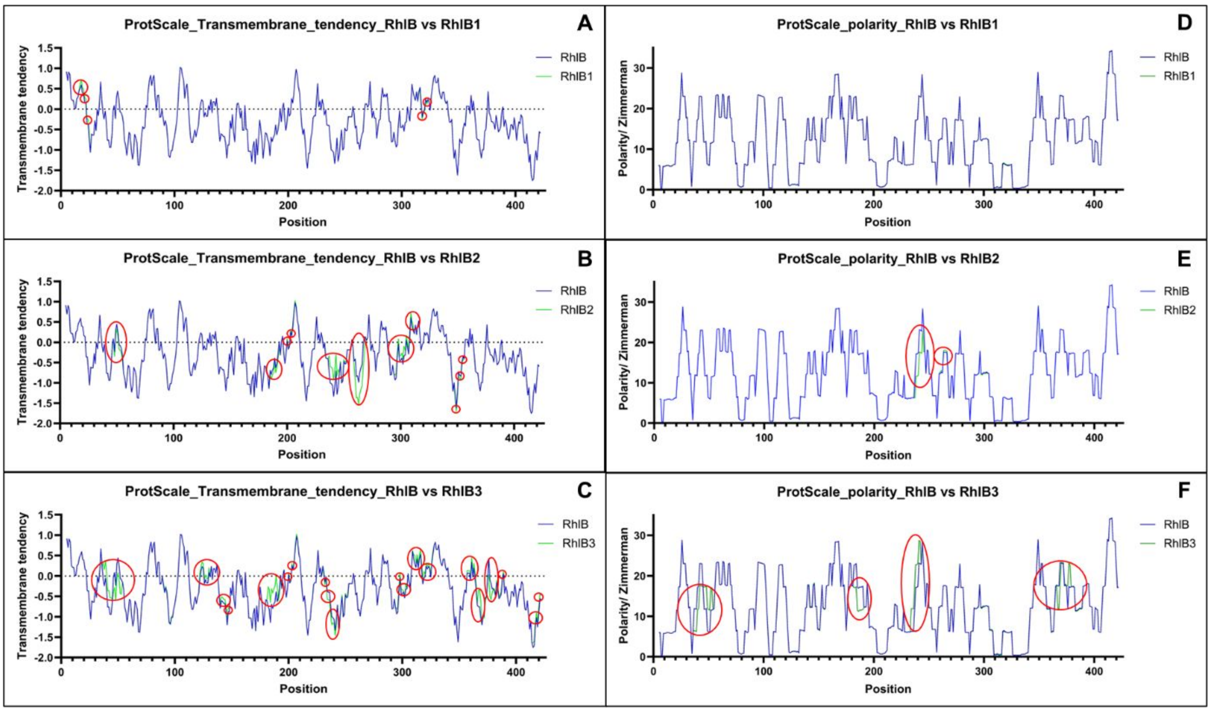

Figure S18. Comparison of transmembrane tendency and polarity profile between the reference RhlB (RhlB_ref) model and the selected RhlB (1, 2, and 3) models. (A–C) Transmembrane tendency comparison using the Zao & London scale. (A) Comparison between RhlB_ref and RhlB1. (B) Comparison between RhlB_ref and RhlB2. (C) Comparison between RhlB_ref and RhlB3. **(**D–F) Polarity profile comparison using the Zimmerman scale. (D) Comparison between RhlB_ref and RhlB1. (E) Comparison between RhlB_ref and RhlB2. (F) Comparison between RhlB_ref and RhlB3. The RhlB_ref model is illustrated using a blue line, while the experimental models are displayed using green. The red circles represent areas where differences between the models appear.

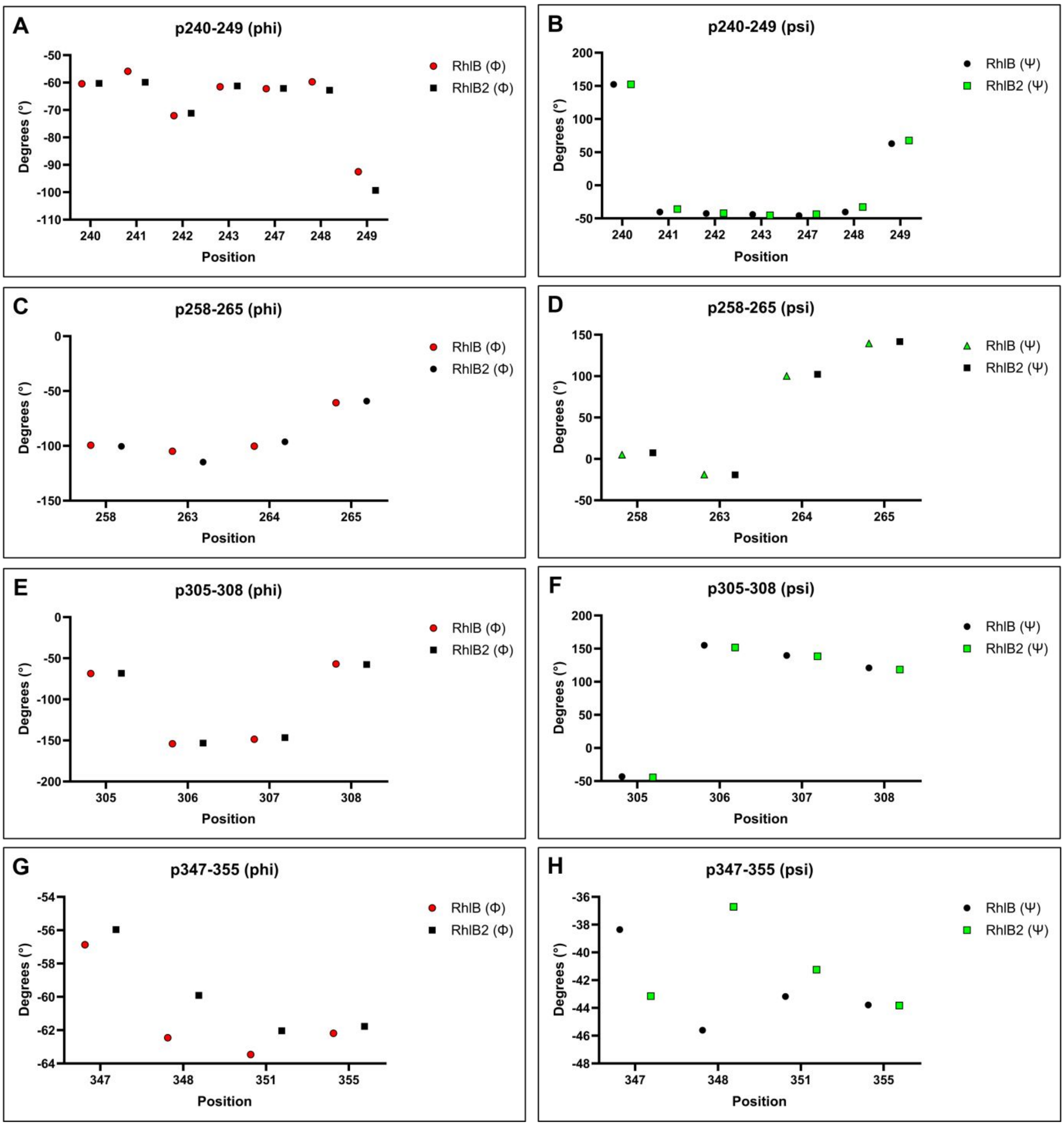

Figure S19. Geometric comparison of reference RhlB and RhlB2. Dihedral angles Φ (phi) and Ψ (psi) between reference RhlB and RhlB2 are compared for residues (A, B) p240-249, (C, D) p258-265, (E, F) p305-308, and (G, H) p347-355. Values are represented in degree from -180º to 180º and were derived from each of the protein model's Ramachandran plots.

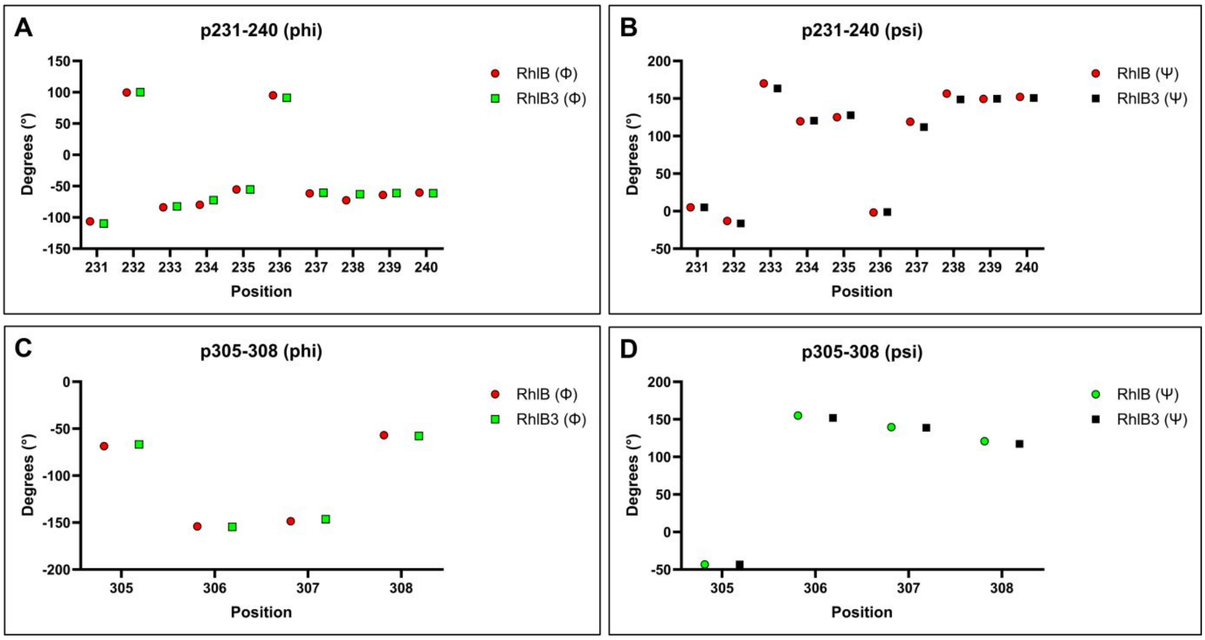

Figure S20. Geometric comparison of reference RhlB and RhlB3. Dihedral angles Φ (phi) and Ψ (psi) between reference RhlB and RhlB2 are compared for residues (A, B) p231-240 and (C, D) p305-308. Values are represented in degree from -180º to 180º and were derived from each of the protein model's Ramachandran plots.

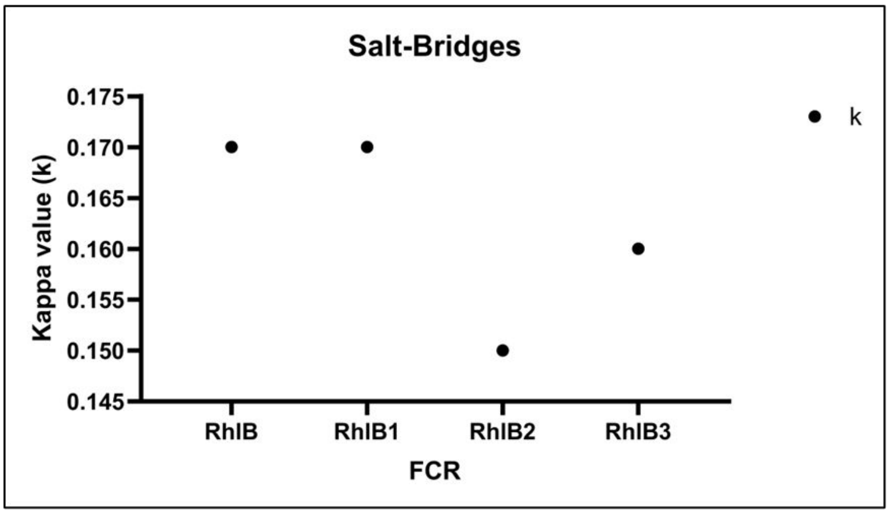

Figure S21. Salt-Bridges comparison between RhlB protein models. The y-axis represents the Kappa value (k) of the protein models, which is a measure of the extent of charge segregation in a protein model. RhlB: 0.17 k, RhlB1: 0.17 k, RhlB2: 0.15 k, RhlB3: 0.16 k. The x-axis illustrates the Fraction of Charged Residues (FCR) value which remained constant between all protein models at 0.19.

Supplementary information for Discussion

RhlB variant Epidemiological distribution

RhlB1

The RhlB1 sequence was derived from the PA TNCF_109 strain isolated from the sputum of a cystic fibrosis (CF) patient at the University of Trento, Italy.[1] The closest phylogenetic protein sequences were clinical isolates from Atlanta Georgia, USA, with three isolates being derived from urine, one from sputum and one from a sacrum ulcer. All sequences shared the mutation L319M located in β11. None of the sequences shared the mutation A21V. Suggesting that the α1 mutation was unique to RhlB1 and the shared β11 mutation could arise naturally in clinical isolates.

RhlB2

The RhlB2 sequence was retrieved from the ROUE01 *P. aeruginosa* strain which was isolated in Besancon, France as part of a study investigating Ceftolozane/ Tazobactam resistance across 420 clinical isolates from 36 public hospitals. Clinical samples were isolated from a range of different infection sources: urine, blood, sputum, wound beds, abscess and internal organs.[2] The ROUE01 strain was isolated from a patient's respiratory tract, however it is not known if it originated from an acute or chronic, a Healthcare-Associated (HCAP) or community acquired pneumonia. An identical sequence was also identified in the *P. aeruginosa* C-18-47465-1-1 strain isolated from a canine auricular infection in Normandy, France.[3,4] The next closest sequence to RhlB2 was identified in the *P. aeruginosa* CMC-115 strain isolated from tracheal aspirate from an elderly male suffering from HCAP in Virginia, USA.[5]

The CMC-115 RhlB sequence differed in two mutations V51A and S315A, which were unique to RhlB2. Identical sequences to the CMC-115 were identified in France and the Netherlands isolated from an expectoration and a CF pulmonary sample respectively. Additionally, similar sequences were isolated from two sputum samples in Pittsburgh and from an Animal-Swine-Sow (caecal) in Wisconsin, USA.

RhlB2 was unable to synthesize M-RL due to a putative ATP-masking mutation at p263. Using H263R as an anchor mutation dysfunctional RhlB sequences were identified across multiple different *P. aeruginosa* strains isolated from both humans and animals particularly swine, equine and canine.[3] The evidence suggests that this *P. aeruginosa* phylogroup evolved in the human pulmonary environment before being transmitted to domesticated animals, with the earliest sequenced RhlB2 variant being isolated from an expectoration sample in France, 1991 (WH-SGI-V-07050). Phylogenetic analysis showed that the RhlB2 variants are distributed primarily across Central and Northern Europe (UK, France, Holland and Germany) and Mid-Atlantic and Midwest, USA (Virginia, Pennsylvania, Wisconsin). The USA isolates could have a transatlantic origin as a result of human or livestock population movements.[6,7]

Schick and Kassen demonstrated that the nutritional availability in the CF pulmonary airway can drive population diversification independently of other factors.[8] Studies have also shown that antimicrobial chemotherapy can modulate bacterial evolution and polymorphic variability.[9,10] Antipseudomonal antibiotics such as ciprofloxacin or aminoglycosides could have led to a hypermutator phenotype emerging, displaying shared mutations in DNA

replication mechanisms.[11] The CMC-115 strain harboured β-lactam, aminoglycoside, vancomycin and chloramphenicol resistant genes.[5] Similarly the ROUE01 strain displayed resistance to extended spectrum β-lactams and β-lactamase inhibitors, 3rd and 4th generation cephalosporins and 2nd generation aminoglycosides.[2] The loss of function observed in RhlB2 is an example of pathway deactivation linked directly to the H263R substitution, making RhlB's ATP-dependent mechanism vulnerable to mutations in resource-limited and antibiotic selective clinical environments.

RhlB3

RhlB3's parental strain originated from an acute otitis externa (*P. paraeruginosa* ATCC 9027), isolated by C. P Hegarty in 1943 in Oregon, USA.[12] To establish an accurate phylogenetic analysis key beneficial mutations (I234V, P238R and S94C) were used as anchor mutations. The closest phylogenetic sequences to RhlB3 were identified in the ZW26, EML1796 and KFSH-Pa27 strains. All three sequences displayed 100% similarity with each other and two mismatches with RhlB3 at p35 and p186, the T35S mutation was unique to RhlB3. All four sequences shared the mutation Q186I but in RhlB3 Glutamine was substituted for Valine.

Epidemiological distribution was interesting with ZW26 being isolated from a CF patient in 1996 in Karlsruhe, Germany, the EML1796 from urban runoff waters in Lyon, France (2015) and KFSH-Pa27 identified as a multi-drug-resistant strain from a tertiary hospital in Saudi Arabia (2023). At first it was believed that the adaptations were a result of hydrocarbon catabolism and external chemical stimuli as all three locations have petrochemical and chemical heavy industries.

However, further analysis identified phylogenetic similar sequences at various sites. The MAZ105 strain was isolated from the rhizosphere of a *Solanum lycopersicum* in Guerrero, Mexico. Similar to the European strains it did not have the T35V mutation, it also showed a unique mutation R181C not present in the RhlB3 sequence. The Maz105 strain was further investigated in a study by Quiroz-Morales that identified an inactivating mutation in *lasR* and *pqsA* affecting the *las* and *pqs* quorum sensing circuits. Consequently, deletions were identified in *rhl*C, *phz*H and type 3 secretion system. The MAZ105 strain demonstrated increased M-RL production in a low phosphate medium, suggesting that low phosphorous levels and a genotypic switch from the central *las* system to *rhl* led to the emergence of a hypermutator phylogroup.[13] It is believed that the Hegarty ATCC 9027 originated from an environmental source either from freshwater or eroded soil, as both can contain limited amounts of phosphorus.[14,15] This was further supported by similar sequences being identified in two *P. aeruginosa* strains; CR1 isolated from chilli rhizosphere in India and A39-1 from a soil sample in China. Furthermore, this strong production phenotype aligns with the need for rhamnolipids as competitive agents in diverse environmental niches.[13] [14,15]

Supplementary information for Methods

Table S3. Bacterial strains, plasmids and constructs used in this study.

Pseudomonas aeruginosa		
**Strain**	**Description**	**Source**
MPAO1	University of Washington subline	Manoil lab, Seattle WA, United states
Escherichia coli		
**Strain**	**Description**	**Source**
DH5α	Cloning strain	Thermo Fisher, UK
**Plasmids**		
**Vectors**	**Description**	**Source**
pUC19	IPTG induction	NEB, UK
pBAD24	Arabinose induction	ATCC
**Constructs**		
**Plasmid**	**Description**	**Source**
pUC19-*rhlAB* (MPAO1)	Native *rhl*AB genes from the MPAO1 subline	This study
pBAD24-*rhlAB*	Reference *rhl*AB genes from the PAO1[16] bioinformatic sequence<br>NCBI accession number: AE004091	This study
pBAD24-*rhlAB*1	Modular construct containing the *rhl*B1 sequence	This study
pBAD24-*rhlAB*2	Modular construct containing the *rhl*B2 sequence	This study
pBAD24-*rhlAB*3	Modular construct containing the *rhl*B3 sequence	This study

Table S4. Nucleotide sequences for reference *rhl*AB in PAO1 (Stover et al., 2000)s

Gene	Nucleotide sequence
rhlA	**ATG**CGGCGCGAAAGTCTGTTGGTATCGGTTTGCAAGGGCCTGCGGGTACATG<br>TCGAGCGCGTTGGGCAGGATCCCGGGCGCAGCACGGTGATGCTGGTCAACGG<br>CGCGATGGCGACCACCGCCTCGTTCGCCCGGACCTGCAAGTGCCTGGCCGAA<br>CATTTCAACGTGGTGCTGTTCGACCTGCCCTTCGCCGGGCAGTCGCGTCAGC<br>ACAACCCGCAGCGCGGGTTGATCACCAAGGACGACGAGGTGGAAATCCTCCT<br>GGCGCTGATCGAGCGCTTCGAGGTCAATCACCTGGTCTCCGCGTCCTGGGGC<br>GGTATCTCCACGCTGCTGGCGCTGTCGCGCAATCCGCGCGGCATCCGCAGCT<br>CGGTGGTGATGGCATTCGCCCCTGGACTGAACCAGGCGATGCTCGACTACGT<br>CGGGCGGGCGCAGGCGCTGATCGAGCTGGACGACAAGTCGGCGATCGGCCAT<br>CTGCTCAACGAGACCGTCGGCAAATACCTGCCGCAGCGCCTGAAAGCCAGCA<br>ACCATCAGCACATGGCTTCGCTGGCCACCGGCGAATACGAGCAGGCGCGCTT<br>TCACATCGACCAGGTGCTGGCGCTCAACGATCGGGGCTACTTGGCTTGCCTG<br>GAGCGGATCCAGAGCCACGTGCATTTCATCAACGGCAGCTGGGACGAATACA<br>CCACCGCCGAGGACGCCCGCCAGTTCCGCGACTACCTGCCGCACTGCAGTTT<br>CTCGCGGGTGGAGGGCACCGGGCATTTCCTCGACCTGGAGTCCAAGCTGGCA<br>GCGGTACGCGTGCACCGCGCCCTGCTCGAGCACCTGCTGAAGCAACCGGAGC<br>CGCAGCGGGCGGAACGCGCGGCGGGATTCCACGAGATGGCCATCGGCTACGC<br>C**TGA**
rhlB	**ATG**CACGCCATCCTCATCGCCATCGGCTCGGCCGGCGACGTATTTCCCTTCA<br>TCGGCCTGGCCCGGACCCTGAAACTGCGCGGGCACCGCGTGAGCCTCTGCAC<br>CATCCCGGTGTTTCGCGACGCGGTGGAGCAGCACGGCATCGCGTTCGTCCCG<br>CTGAGCGACGAACTGACCTACCGCCGGACCATGGGCGATCCGCGCCTGTGGG<br>ACCCCAAGACGTCCTTCGGCGTGCTCTGGCAAGCCATCGCCGGGATGATCGA<br>GCCGGTCTACGAGTACGTCTCGGCGCAGCGCCATGACGACATCGTGGTGGTC<br>GGCTCGCTATGGGCGCTGGGCGCACGCATCGCTCACGAGAAGTACGGGATTC<br>CCTACCTGTCCGCGCAGGTCTCGCCATCGACCCTGTTGTCGGCGCACCTGCC<br>GCCGGTACACCCCAAGTTCAACGTGCCCGAGCAGATGCCGCTGGCGATGCGC<br>AAGCTGCTCTGGCGCTGCATCGAGCGCTTCAAGCTGGATCGCACCTGCGCGC<br>CGGAGATCAACGCGGTGCGCCGCAAGGTCGGCCTGGAAACGCCGGTGAAGCG<br>CATCTTCACCCAATGGATGCATTCGCCGCAGGGCGTGGTCTGCCTGTTCCCG<br>GCCTGGTTCGCGCCGCCCCAGCAGGATTGGCCGCAACCCCTGCACATGACCG<br>GCTTCCCGCTGTTCGACGGCAGTATCCCGGGGACCCCGCTCGACGACGAACT<br>GCAACGCTTTCTCGATCAGGGCAGCCGGCCGCTGGTGTTCACCCAGGGCTCG<br>ACCGAACACCTGCAGGGCGACTTCTACGCCATGGCCCTGCGCGCGCTGGAAC<br>GCCTCGGCGCGCGTGGGATCTTCCTCACCGGCGCCGGCCAGGAACCGCTGCG<br>CGGCTTGCCGAACCACGTGCTGCAGCGCGCCTACGCGCCACTGGGAGCCTTG<br>CTGCCATCGTGCGCCGGGCTGGTCCATCCGGGCGGTATCGGCGCCATGAGCC<br>TAGCCTTGGCGGCGGGGGTGCCGCAGGTGCTGCTGCCCTGTGCCCACGACCA<br>GTTCGACAATGCCGAACGGCTGGTCCGGCTCGGCTGCGGGATGCGCCTGGGC<br>GTGCCGTTGCGCGAGCAGGAGTTGCGCGGGGCGCTGTGGCGCTTGCTCGAGG<br>ACCCGGCCATGGCGGCGGCCTGTCGGCGTTTCATGGAATTGTCACAACCGCA<br>CAGTATCGCTTGCGGTAAAGCGGCCCAGGTGGTCGAACGTTGTCATAGGGAG<br>GGGGATGCTCGATGGCTGAAGGCTGCGTCC**TGA**

Table S5. Active domains at the nucleotide and protein level

Gene ID	Nucleotide Active domains		Protein Active domains	
rhlB	Envelope: 7 – 414	Alignment: 10 – 405	Envelope: 3 – 138	Alignment: 4 – 135
	Envelope: 607 – 1221	Alignment: 691 – 1141	Envelope: 203 – 407	Alignment: 231 – 397
***Note:** Nucleotide and protein active domains correspond to nucleotide and amino acid positions in the reference *rhlB*/RhlB sequence (PAO1, Stover et al., 2000).				

Table S6. Reference RhlB primary structure parameters

Type of turn	Amino acid sequence	position
α-turn (4 aa)	QHGI, SLWA, AYAP, AGVP, LSQP	p45-48, p106-109, p305-308, p334-337, p395-398
β-turn (3 aa)	RGH, LSD, GIP, GAR, PGG	p27-29, p53-55, p120-122, p280-282, p322-324,
γ-turn (2 aa)	GD, VS, CA, GC, ED	p64-65, p128-129, p316-317, p358-359, p381-382
δ-turn (1 aa)	I	p36
π-turn (5 aa)	WDPKT, QRHDD, QGSRP	p69-73, p96-100, p249-253
**Type of loop**	**Amino acid sequence**	**position**
Short (4-7 aa)	AIGSAGD, MHSPQG, LPCAHD, LGVPLR	P7-13, p197-202, p341-346, p363-368
Medium (8-12 aa)	GLETPVKR, QGSTEHLQG	p184-191, p258-266
Long (13-20 aa)	LFPAWFAPPQQDWPQP, TGFPLFDGSIPGTPLD, TGAGQEPLRGLPNH	P206-221, p225-240, p286-300
Very long (over 20 aa)	LLSAHLPPVHPKFNVPEQMP	p133-152

Table S7. Reference RhlB secondary structure parameters

**RhlB reference sequence: PAO1 Stover et al., 2000**		
M**HAILI**AIGSAGD**VFPFIGLARTLKL**RGH**RVSLCT**I**PVFRDAVE**QHGI**AFVP**LSD**ELTYRRTM**GD**PRL**WDPKT<br>**SFGVLWQAIAGMIEPVYEYVSA**QRHDD**IVVVG**SLWA**LGARIAHEKY**GIP**YLSAQ**VS**PST**LLSAHLPPVHPKFN<br>VPEQMP**LAMRKLLWRCIERFKLDRTCAPEINAVRRKV**GLETPVKR**IFTQW**MHSPQG**VVC**LFPAWFAPPQQDWP<br>QP**LHM**TGFPLFDGSIPGTPLD**DELQRFLD**QGSRP**LVFT**QGSTEHLQG**DFYAMALRALERL**GAR**GIFL**TGAGQE<br>PLRGLPNH**VLQR**AYAP**LGALLPS**CA**GLVH**PGG**IGAMSLALA**AGVP**QVL**LPCAHD**QFDNAERLVRL**GC**GMR**LGV<br>PLR**EQELRGALWRLL**ED**PAMAAACRRFMEL**SQP**HSIACGKAAQVVERCHREGDARW**LKAAS		
**Type of secondary structure**	**Amino acid sequence**	**Amino acid position**
	**α-helix**	
1st α-helix	**VFPFIGLARTLKL**	p14-26
2nd α-helix	**PVFRDAVE**	p37-44
3rd α-helix	**ELTYRRTM**	p56-63
4th α-helix	**PRL**	p66-68
5th α-helix	**SFGVLWQAIAGMIEPVYEYVSA**	p74-95
6th α-helix	**LGARIAHEKY**	p110-119
7th α-helix	**PST**	p130-132
8th α-helix	**LAMRKLLWRCIERFKLDRTCAPEINAVRRKV**	p153-183
9th α-helix	**IFTQW**	p192-196
10th α-helix	**DELQRFLD**	p241-248
11th α-helix	**DFYAMALRALERL**	p267-279
12th α-helix	**LGALLPS**	p309-315
13th α-helix	**IGAMSLALA**	p325-333
14th α-helix	**QFDNAERLVRL**	p347-357
15th α-helix	**EQELRGALWRLL**	p369-380
16th α-helix	**PAMAAACRRFME**	p383-394
17th α-helix	**HSIACGKAAQVVERCHREGDARW**	p399-421
	**β-sheet**	
1st β-sheet	**HAILI**	p2-6
2nd β-sheet	**RVSLCT**	p30-35
3rd β-sheet	**AFVP**	p49-52
4th β-sheet	**IVVVG**	p101-105
5th β-sheet	**YLSAQ**	p123-127
6th β-sheet	**VVC**	p203-205
7th β-sheet	**LHM**	p222-224
8th β-sheet	**LVFT**	p254-257
9th β-sheet	**GIFL**	p283-286
10th β-sheet	**VLQR**	p301-304
11th β-sheet	**GLVH**	p318-321
12th β-sheet	**QVL**	p338-340
13th β-sheet	**GMR**	p360-362

Table S8. SMILES notations for Ligands used for small molecule docking

Ligand	SMILES
HAAs	[CCCCCCCC(CC(=O)O)O]
M-RL	[CCCCCCCC(CC(=O)O)OC(=O)CC(CCCCCCC)O[C@H]1[C@@H]([C@@H]([C@H]([C@@H](O1)C)O)O)O]
dTDP-L-rhamnose	[C[C@H]1[C@@H]([C@H]([C@H](C(O1)OP(=O)(O)OP(=O)(O)OC[C@@H]2[C@H](C[C@@H](O2)N3C=C(C(=O)NC3=O)C)O)O)O)O]
ATP	[C1=NC(=C2C(=N1)N(C=N2)[C@H]3[C@@H]([C@@H]([C@H](O3)COP(=O)(O)OP(=O)(O)OP(=O)(O)O)O)O)N]
GTP	[C1=NC2=C(N1[C@H]3[C@@H]([C@@H]([C@H](O3)COP(=O)(O)OP(=O)(O)OP(=O)(O)O)O)O)N=C(NC2=O)N]

Table S9. RhlB candidate protein sequences. (Substitutions from reference sequence in yellow)

Sequence ID	Protein sequence
Reference RhlB	MHAILIAIGSAGDVFPFIGLARTLKLRGHRVSLCTIPVFRDAVEQHGI<br>AFVPLSDELTYRRTMGDPRLWDPKTSFGVLWQAIAGMIEPVYEYVSAQ<br>RHDDIVVVGSLWALGARIAHEKYGIPYLSAQVSPSTLLSAHLPPVHPK<br>FNVPEQMPLAMRKLLWRCIERFKLDRTCAPEINAVRRKVGLETPVKRI<br>FTQWMHSPQGVVCLFPAWFAPPQQDWPQPLHMTGFPLFDGSIPGTPLD<br>DELQRFLDQGSRPLVFTQGSTEHLQGDFYAMALRALERLGARGIFLTG<br>AGQEPLRGLPNHVLQRAYAPLGALLPSCAGLVHPGGIGAMSLALAAGV<br>PQVLLPCAHDQFDNAERLVRLGCGMRLGVPLREQELRGALWRLLEDPA<br>MAAACRRFMELSQPHSIACGKAAQVVERCHREGDARWLKAAS
RhlB1	MHAILIAIGSAGDVFPFIGLVRTLKLRGHRVSLCTIPVFRDAVEQHGI<br>AFVPLSDELTYRRTMGDPRLWDPKTSFGVLWQAIAGMIEPVYEYVSAQ<br>RHDDIVVVGSLWALGARIAHEKYGIPYLSAQVSPSTLLSAHLPPVHPK<br>FNVPEQMPLAMRKLLWRCIERFKLDRTCAPEINAVRRKVGLETPVKRI<br>FTQWMHSPQGVVCLFPAWFAPPQQDWPQPLHMTGFPLFDGSIPGTPLD<br>DELQRFLDQGSRPLVFTQGSTEHLQGDFYAMALRALERLGARGIFLTG<br>AGQEPLRGLPNHVLQRAYAPLGALLPSCAGMVHPGGIGAMSLALAAGV<br>PQVLLPCAHDQFDNAERLVRLGCGMRLGVPLREQELRGALWRLLEDPA<br>MAAACRRFMELSQPHSIACGKAAQVVERCHREGDARWLKAAS
RhlB2	MHAILIAIGSAGDVFPFIGLARTLKLRGHRVSLCTIPVFRDAVEQHGI<br>AFAPLSDELTYRRTMGDPRLWDPKTSFGVLWQAIAGMIEPVYEYVSAQ<br>RHDDIVVVGSLWALGARIAHEKYGIPYLSAQVSPSTLLSAHLPPVHPK<br>FNVPEQMPLAMRKLLWRCIERFKLDRTCAPEINAVRRKVGLETPAKRI<br>FTQWMHSPQGVLCLFPAWFAPPQQDWPQPLHMTGFPLFDGSIPGTPLD<br>AELQRFLEQGSRPLVFTQGSTERQQGDFYAMALRALERLGARGIFLTG<br>AGQEPLRGLPSHVLQRAYVPLGALLPSCAGLVHPGGIGAMSLALAAGV<br>PQVLLPCAHDQFDNTERLVRLGCGMRLGVPLREQELRGALWRLLEDPA<br>MAAACRRFMELSQPHSIACGKAAQVVERCHREGDARWLKAAS
RhlB3	MHAILIAIGSAGDVFPFIGLARTLKLRGHRVSLCSIPVFRAAVEQHGI<br>EFVPLSDELTYRRTMGDPRLWDPKTSFGVLWQAIAGMIEPVYEYVCAQ<br>RHDDIVVVGSLWALGARIAHEKYGIPYLSVQVSPSTLLSAHLPPVHPR<br>FNVPEQVPLAMRKLLWRCIERFKLDRTCAPEINAVRRKVGLVGPAKRI<br>FTQWMHSPQGVLCLFPAWFAPPQQDWPQPLHMTGFPLFDGSVPGTRLD<br>DELQRFLEQGSRPLVFTQGSTEHLQGDFYAMALRALERLGARGIFLTG<br>AGQEPLRGLPSHVLQRSYVPLGALLPACAGLVHPAGIGAMSLALAAGV<br>PQVLLPCAHDQFDNAERLVRLGCGIRLGLPLREQALRESLWRLLEDPA<br>LAAACRRFMELSQPHSIACGKAAQVVERCHREGDVRWLKAAS

Table S10. Composition of *E. coli* induction media for rhamnolipid production optimisation

Components	Concentration	Media 1	Media 2	Media 3	Media 4	Media 5	Media 6
Sucrose	10%	1 ml	1 ml	-	1 ml	1 ml	-
Glucose	10%	-	-	-	-	-	1 ml
L-rhamnose	10%	-	-	1 ml	-	-	-
Glycerol	10%	1 ml	1 ml	1 ml	1 ml	1 ml	1 ml
Base media M9 salts	1x	7.5 ml	7.5 ml	7.5 ml	7.5 ml	7.5 ml	7.5 ml
Peptone	100 mg	-	-	-	0.5 ml	-	-
Yeast extract	5 mg	-	-	-	-	0.5 ml	-
Tryptone	5%	-	0.5 ml	-	-	-	-
Casamino acids	5%	0.5 ml	-	0.5 ml	-	0.5 ml	0.5 ml
IPTG	0.4 mM	50ul	50ul	50ul	50ul	50ul	50ul
Carbenicillin	50 ug/ml	10ul	10ul	10ul	10ul	10ul	10ul
**Total volume**	**10 ml**						

Table S11. Composition of *E. coli* Screening Media (ESM)

Media components	ESM
10% Glycerol	1 ml
1M Sucrose	1 ml
20% w/v Casamino acids	0.5 ml
Yeast extract	0.5 ml
NaCl	0.2 ml
Carbenicillin (50 µg ml^{-1})	10 µl
Bacterial inoculum in PBS (OD_{600}:0.1)	0.1 ml
M9 salts	-
Autoclaved dH2O	6.7 ml
**Total volume**	**10 ml**

Table S12. *rhlA*B PCR and Sequencing primers

Primer	Vector/ Strain	Nucleotide sequence	Restriction enzyme
Forward *rhlA*	pBAD24	5'-ATATA**GAATTC**ATGCGGCGCGAAAG-3'	*EcoRI*
Forward *rhlA*	pUC19	5'-ATATA**GCATGC**ATGCGGCGCGAAAG-3'	*SphI*
Reverse *rhlA*	pBAD24	5'-GT**GGTACC**TCAGGCGTAGCCGATG-3'	*KpnI*
Reverse *rhlB*	pBAD24	5'-CG**AGCATGC**TCAGGACGCAGC-3'	*SphI*
Reverse *rhlB*	pUC19	5'-GCT**GAGCTC**TCAGGACGCAGCCTTC-3'	*SacI*
**Sanger sequencing screening primers**			
Forward MCS	pBAD24	5'-CTCCATACCCGTTTTTTTGGGC-3'	N/A
Reverse MCS	pBAD24	5'-GCTGAAAATCTTCTCTCATCCG-3'	N/A
M13 Forward (-21)	pUC19	5'-TGTAAAACGACGGCCAGT-3'	N/A
M13 Forward (-43)	pUC19	5'-AGGGTTTTCCCAGTCACGACGTT-3'	N/A
M13 Reverse (-29)	pUC19	5'-CAGGAAACAGCTATGACC-3'	N/A
M13 Reverse (-49)	pUC19	5'-GAGCGGATAACAATTTCACACAGG-3'	N/A
Reverse *rhlA*	pBAD24 + pUC19	5'-CGCAGGTCAAGGGTTCAGGC-3'	N/A
Forward *rhlB*	pBAD24 + pUC19	5'-GCATAACGCACGGAGTAGCCCC-3'	N/A
Reverse *rhlB*	pBAD24 + pUC19	5'-CCGGATTCAGGACGCAGCC-3'	N/A
**qPCR primers**			
Forward 16S	*E. coli*	5'-GTCAGCTCGTGTTGTGAAATG-3'	N/A
Reverse 16S	*E. coli*	5'-CCCACCTTCCTCCAGTTTATC-3'	N/A
Forward *rhlB*	pBAD24	5'-GTAGGTCAGTTCGTCGCTCA-3'	N/A
Reverse *rhlB*	pBAD24	5'-CGACGTATTTCCCTTCATCGG-3'	N/A

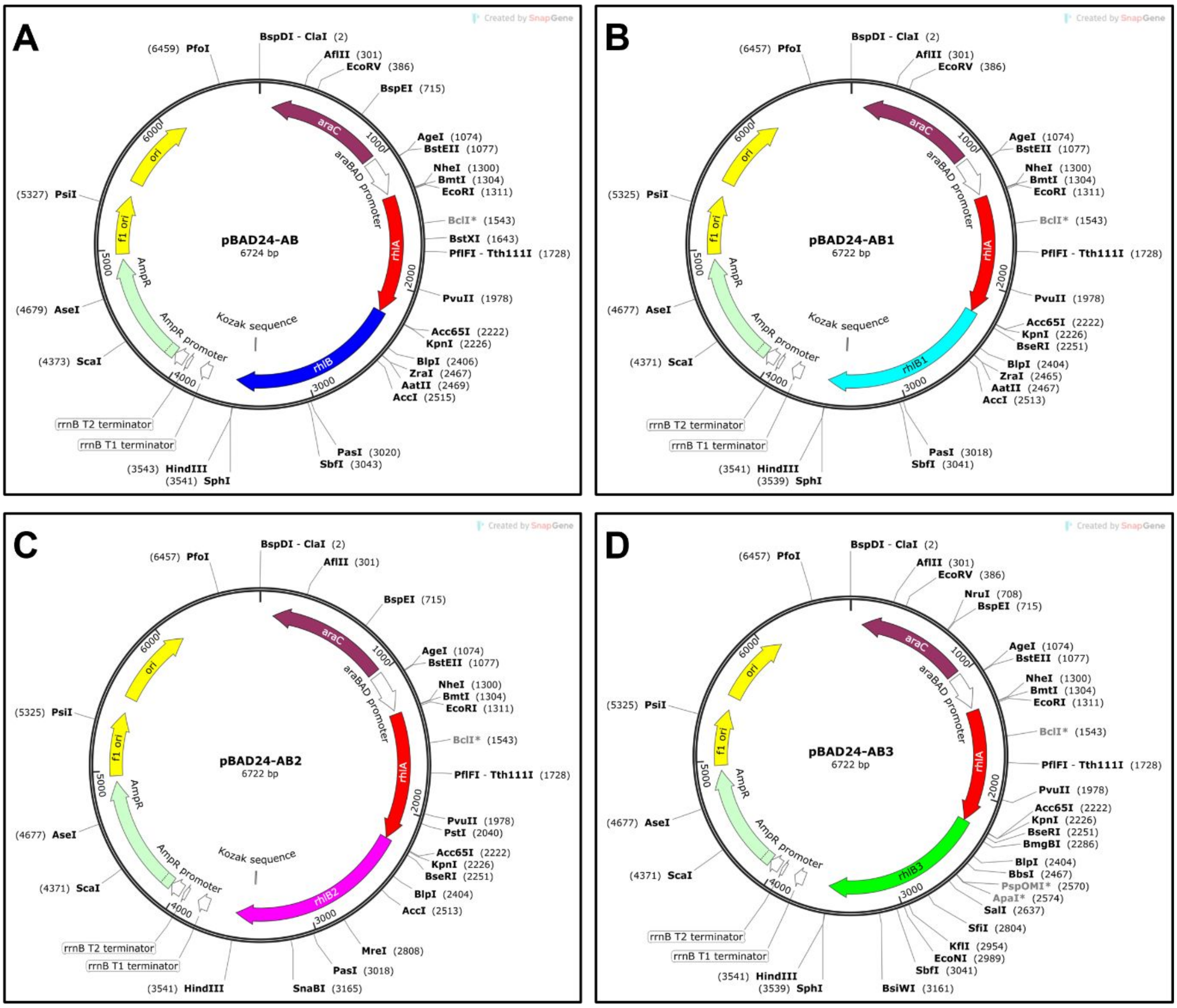

Figure S22. Mono-rhamnolipid pathway plasmid maps. (A) pBAD24-*rhlAB* construct (contains reference *rhlA* and *rhlB*). (B) pBAD24-*rhlAB*1 construct (reference *rhlA* and *rhlB1* gene variant). (C) pBAD24-*rhlAB*2 construct (reference *rhlA* and *rhlB2* gene variant). (D) pBAD24-*rhlAB*3 construct (reference *rhlA* and *rhlB3* gene variant). All plasmid maps were created using SnapGene.

References

(1) Bianconi, I.; D'Arcangelo, S.; Benedet, M.; Bailey, K. E.; Esposito, A.; Piffer, E.; Mariotto, A.; Baldo, E.; Dinnella, G.; Gualdi, P.; Schinella, M.; Donati, C.; Jousson, O. Draft Genome Sequences of 40 Pseudomonas Aeruginosa Clinical Strains Isolated from the Sputum of a Single Cystic Fibrosis Patient Over an 8-Year Period. *Genome Announc.* **2016**, *4* (6), e01205-16. https://doi.org/10.1128/genomeA.01205-16.

(2) Fournier, D.; Carrière, R.; Bour, M.; Grisot, E.; Triponney, P.; Muller, C.; Lemoine, J.; Jeannot, K.; Plésiat, P. Mechanisms of Resistance to Ceftolozane/Tazobactam in Pseudomonas Aeruginosa: Results of the GERPA Multicenter Study. *Antimicrob. Agents Chemother.* **2021**, *65* (2), e01117-20. https://doi.org/10.1128/AAC.01117-20.

(3) Pottier, M.; Gravey, F.; Castagnet, S.; Auzou, M.; Langlois, B.; Guérin, F.; Giard, J.-C.; Léon, A.; Le Hello, S. A 10-Year Microbiological Study of Pseudomonas Aeruginosa Strains Revealed the Circulation of Populations Resistant to Both Carbapenems and Quaternary Ammonium Compounds. *Sci. Rep.* **2023**, *13* (1), 2639. https://doi.org/10.1038/s41598-023-29590-0.

(4) Pottier, M.; Castagnet, S.; Gravey, F.; Leduc, G.; Sévin, C.; Petry, S.; Giard, J.-C.; Le Hello, S.; Léon, A. Antimicrobial Resistance and Genetic Diversity of Pseudomonas Aeruginosa Strains Isolated from Equine and Other Veterinary Samples. *Pathogens* **2022**, *12* (1), 64. https://doi.org/10.3390/pathogens12010064.

(5) Adenikinju, A.; Jensen, R. V.; Kerkering, T. M.; Garner, D. C.; Rao, J. Complete Genome Sequence of Pseudomonas Aeruginosa CMC-115, a Clinical Strain from an Acute Ventilator-Associated Pneumonia Patient. *Microbiol. Resour. Announc.* **2020**, *9* (30), e00595-20. https://doi.org/10.1128/MRA.00595-20.

(6) Aagaard-Hansen, J.; Nombela, N.; Alvar, J. Population Movement: A Key Factor in the Epidemiology of Neglected Tropical Diseases. *Trop. Med. Int. Health* **2010**, *15* (11), 1281–1288. https://doi.org/10.1111/j.1365-3156.2010.02629.x.

(7) Castañeda-Barba, S.; Top, E. M.; Stalder, T. Plasmids, a Molecular Cornerstone of Antimicrobial Resistance in the One Health Era. *Nat. Rev. Microbiol.* **2024**, *22* (1), 18–32. https://doi.org/10.1038/s41579-023-00926-x.

(8) Schick, A.; Kassen, R. Rapid Diversification of *Pseudomonas Aeruginosa* in Cystic Fibrosis Lung-like Conditions. *Proc. Natl. Acad. Sci.* **2018**, *115* (42), 10714–10719. https://doi.org/10.1073/pnas.1721270115.

(9) Gustafsson, I.; Sjölund, M.; Torell, E.; Johannesson, M.; Engstrand, L.; Cars, O.; Andersson, D. I. Bacteria with Increased Mutation Frequency and Antibiotic Resistance Are Enriched in the Commensal Flora of Patients with High Antibiotic Usage. *J. Antimicrob. Chemother.* **2003**, *52* (4), 645–650. https://doi.org/10.1093/jac/dkg427.

(10) Revitt-Mills, S. A.; Robinson, A. Antibiotic-Induced Mutagenesis: Under the Microscope. *Front. Microbiol.* **2020**, *11*. https://doi.org/10.3389/fmicb.2020.585175.

(11) Garushyants, S. K.; Sane, M.; Selifanova, M. V.; Agashe, D.; Bazykin, G. A.; Gelfand, M. S. Mutational Signatures in Wild Type Escherichia Coli Strains Reveal Predominance of DNA Polymerase Errors. *Genome Biol. Evol.* **2024**, *16* (4), evae035. https://doi.org/10.1093/gbe/evae035.

(12) Mai-Prochnow, A.; Bradbury, M.; Murphy, A. B. Draft Genome Sequence of Pseudomonas Aeruginosa ATCC 9027 (DSM 1128), an Important Rhamnolipid Surfactant Producer and Sterility Testing Strain. *Genome Announc.* **2015**, *3* (5), e01259-15. https://doi.org/10.1128/genomeA.01259-15.

(13) Quiroz-Morales, S. E.; Muriel-Millán, L. F.; Ponce-Soto, G. Y.; González-Valdez, A.; Castillo-Juárez, I.; Servín-González, L.; Soberón-Chávez, G. Pseudomonas Aeruginosa Strains Belonging to Phylogroup 3 Frequently Exhibit an Atypical Quorum Sensing Response: The Case of MAZ105, a Tomato Rhizosphere Isolate. *Microbiology* **2023**, *169* (10), 001401. https://doi.org/10.1099/mic.0.001401.

(14) Xiaofei Ma; Chengyi Zhao; Jianting Zhu. Aggravated Risk of Soil Erosion with Global Warming – A Global Meta-Analysis. *CATENA* **2021**, *200*, 105129. https://doi.org/10.1016/j.catena.2020.105129.

(15) Elser, J. J.; Bracken, M. E. S.; Cleland, E. E.; Gruner, D. S.; Harpole, W. S.; Hillebrand, H.; Ngai, J. T.; Seabloom, E. W.; Shurin, J. B.; Smith, J. E. Global Analysis of Nitrogen and Phosphorus Limitation of Primary Producers in Freshwater, Marine and Terrestrial Ecosystems. *Ecol. Lett.* **2007**, *10* (12), 1135–1142. https://doi.org/10.1111/j.1461-0248.2007.01113.x.

(16) Stover, C. K.; Pham, X. Q.; Erwin, A. L.; Mizoguchi, S. D.; Warrener, P.; Hickey, M. J.; Brinkman, F. S. L.; Hufnagle, W. O.; Kowalik, D. J.; Lagrou, M.; Garber, R. L.; Goltry, L.; Tolentino, E.; Westbrock-Wadman, S.; Yuan, Y.; Brody, L. L.; Coulter, S. N.; Folger, K. R.; Kas, A.; Larbig, K.; Lim, R.; Smith, K.; Spencer, D.; Olson, M. V. PAO1, an Opportunistic Pathogen. *Nature* **2000**, *406*, 959-964.
